# Evaluation of Nanopore Sensor Design Using Electrical and Optical Analyses

**DOI:** 10.1021/acsnano.3c02532

**Published:** 2023-06-01

**Authors:** Lauren A. Mayse, Ali Imran, Yazheng Wang, Mohammad Ahmad, Rebecca A. Oot, Stephan Wilkens, Liviu Movileanu

**Affiliations:** † Department of Physics, Syracuse University, 201 Physics Building, Syracuse, New York 13244-1130, United States; ‡ Department of Biomedical and Chemical Engineering, Syracuse University, 329 Link Hall, Syracuse, New York 13244, United States; § Department of Biochemistry and Molecular Biology, State University of New York-Upstate Medical University, 750 East Adams Street, Syracuse, New York 13210, United States; ∥ The BioInspired Institute, Syracuse University, Syracuse, New York 13244, United States

**Keywords:** protein engineering, nanosensor, nanodisc, single-molecule electrophysiology, biolayer interferometry, real-time kinetics, protein detection

## Abstract

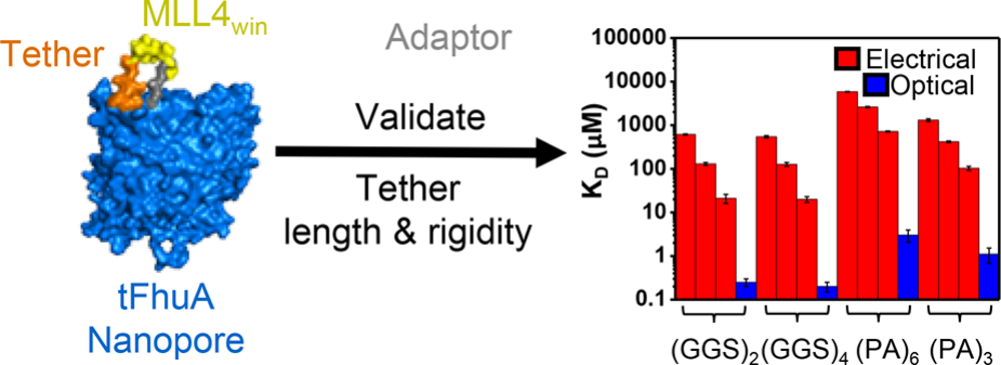

Nanopores are currently utilized as powerful tools for single-molecule protein sensing. The reporting signal typically requires protein analytes to enter the nanopore interior, yet a class of these sensors has emerged that allows targeted detection free in solution. This tactic eliminates the spatial limitation of nanopore confinement. However, probing proteins outside the nanopore implies numerous challenges associated with transducing the physical interactions in the aqueous phase into a reliable electrical signature. Hence, it necessitates extensive engineering and tedious optimization routes. These obstacles have prevented the widespread adoption of these sensors. Here, we provide an experimental strategy by developing and validating single-polypeptide-chain nanopores amenable to single-molecule and bulk-phase protein detection approaches. We utilize protein engineering, as well as nanopore and nanodisc technologies, to create nanopore sensors that can be integrated with an optical platform in addition to traditional electrical recordings. Using the optical modality over an ensemble of detectors accelerates these sensors’ optimization process for a specific task. It also provides insights into how the construction of these single-molecule nanopore sensors influences their performance. These outcomes form a basis for evaluating engineered nanopores beyond the fundamental limits of the resistive-pulse technique.

A single nanopore is a versatile sensing element for numerous tasks in protein analytics.^1−6^ Significant progress has been accomplished in basic research and biosensing technology using nanopores^7−9^ fabricated in various scaffolds and materials.^10−15^ The readout signal in these sensors is the transmembrane current through a nanopore.^16^ Key advantages that make this approach influential include the following: (i) this label-free method probes time-resolved molecular events at a single-molecule level;^17−19^ (ii) the nanopore structure and composition can be altered with atomic precision;^9,20^ (iii) nanopores are amenable to automated microelectrode recording technologies;^21−23^ (iv) electrical recordings with single nanopores can be conducted in a broad dynamic range of interactions and analyte concentrations;^24^ (v) specific and sensitive detection can be performed in challenging heterogeneous solutions, such as biofluids,^25−28^ or in complex mixtures of proteins.^29^ Therefore, this approach shows promise in the wide-time bandwidth evaluation of single-protein dynamics. Nanopore sensors can also illuminate numerous structural and functional characteristics of proteins, including their shape and size,^30^ enzymatic activity,^31−34^ mechanical stability,^6,18,35^ cotranslocational unfolding,^35−38^ and post-translational modifications.^39−43^ For example, a significant benefit is an ability to unravel dynamic fluctuations of protein sizes and conformations in solution using glass^44^ and solid-state nanopores.^10,30,45,46^ In addition, nanopores are nowadays utilized to conduct peptide and protein profiling.^47−49^ More recently, several studies showed prospects of nanopores in single-molecule protein sequencing.^50−53^

With several exceptions,^28,29,54−59^ the output signal necessitates the target protein to navigate into the nanopore lumen for further analysis. This impairs the ability to monitor large proteins that cannot enter the nanopore. Further, detecting protein receptor–protein ligand interactions outside the nanopore can resemble a more realistic interaction that occurs in nature. Yet, sensing this physical process can be accomplished by tethering a single protein recognition element to the nanopore. There has been substantial advancement in the creation of these sensors, but it is apparent that they come with persistent obstacles. The most intimidating difficulty is a mechanistic understanding of how these sensors transduce the physical interactions in the aqueous phase into a reliable electrical signature without perturbing the resulting single-molecule kinetics. For example, even if these challenges are addressed, it needs to be clarified what are the implications of the restraint of the tethered protein recognition element on the frequency and duration of target protein captures in the solution. This question is motivated by experimental evidence showing that the kinetics^60,61^ and dynamics^62−65^ of protein recognition depend on the flexibility and length of the tether, which immobilizes one binding partner to a surface. However, most of the literature pertaining to this topic comes from computational^66−68^ and theoretical^69−73^ studies. Furthermore, we highlight that in many cases evaluating these sensors’ performance solely relies on the resistive-pulse technique (Figure 1a), limiting our knowledge of their quantitative and functional traits. Therefore, there is a pressing demand for a direct confirmatory method, which should have the following attributes: (i) it employs an alternative readout, (ii) it preserves the nanopore’s architecture, (iii) it provides real-time kinetics of protein captures, and (iv) it has potential for acquiring data in a scalable setting.

**Figure 1:**
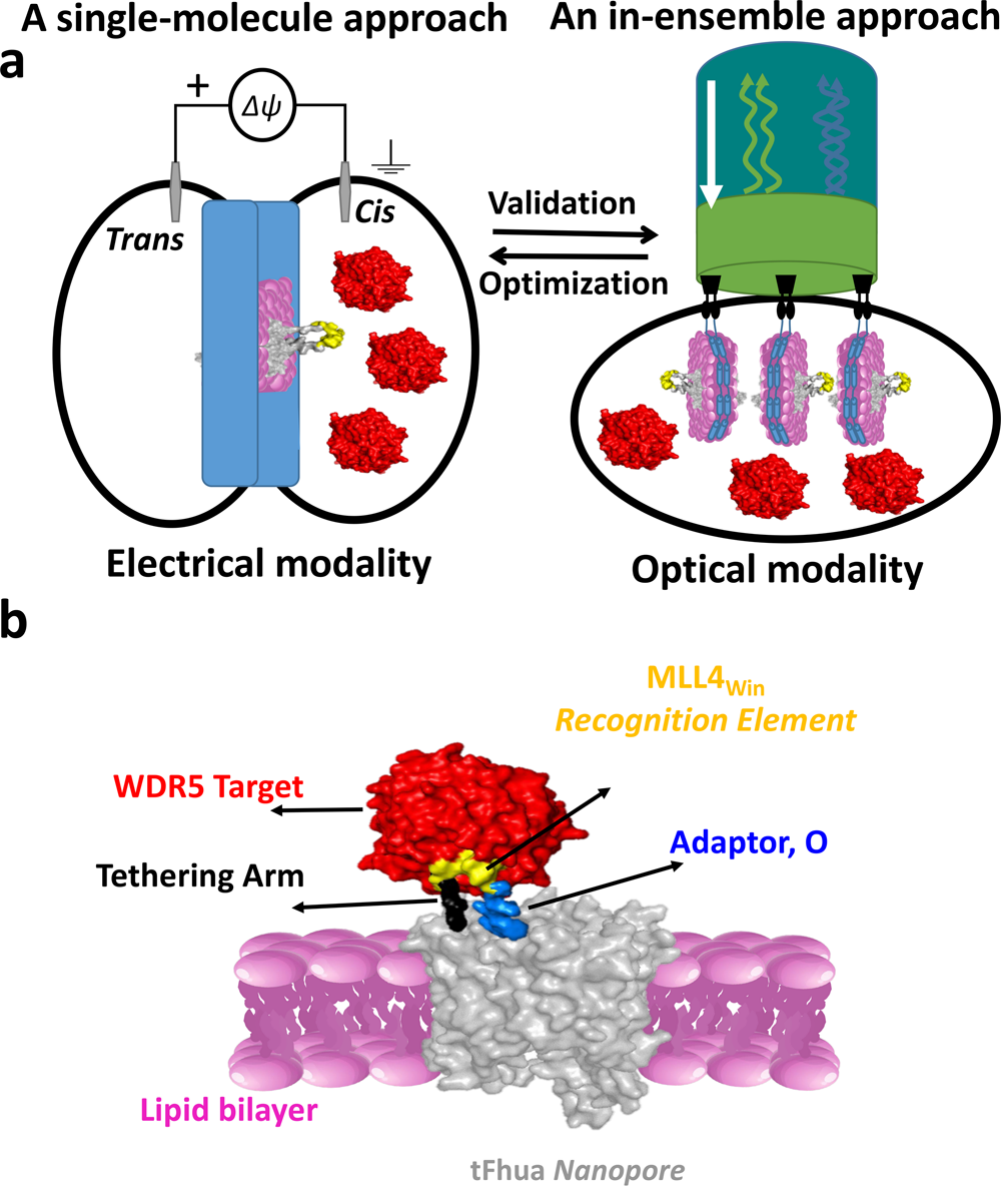
Engineered nanopores for two protein detection modalities. (a) On the left side, this graphic shows a nanopore sensor amenable to a single-molecule protein detection modality. This nanopore (gray), which features a protein recognition element (yellow), is reconstituted into a lipid bilayer (magenta) supported by a Teflon partition (blue). The target protein (red), WDR5, is added to the *cis* side. Time-resolved single-channel electrical recordings can be conducted using this sensor formulation. On the right side, this panel illustrates a nanopore sensor amenable to a bulk-phase protein detection modality. This nanopore is immobilized on the surface of a biolayer interferometry (BLI) sensing chip for optical determinations. In addition, this nanopore sensor is amalgamated with synthetic lipids (magenta) enclosed by two membrane scaffold proteins (MSPs; in blue) to form a nanodisc. The nanodisc was then biotinylated and attached to the streptavidin (SA)-coated BLI sensor (in black). A white light is then directed to the BLI sensor and the target protein (red), WDR5, is added to an aqueous well in which the sensor is dipped. (b) A nanopore equipped with a 14-residue mixed-lineage leukemia (MLL4_Win_) Win motif ligand. This sensor has four exchangeable elements: a tFhuA protein pore (gray), a tethering arm (black), a protein recognition element (yellow), and a peptide adaptor (blue).

To address this technological gap, we adapted a nanopore sensor to the biolayer interferometry (BLI) platform (Figure 1a).^74^ This biosensing technology monitors the accumulation of immobilized ligand–protein complexes through alterations in the interference pattern between reflected light waves at the surface of the BLI sensor. The primary reason for this choice is that such a technology can probe real-time and label-free protein kinetics. In addition, we utilized the nanodisc (ND) technology^75^ to optimize the BLI performance and provide the lipid membrane surroundings.^76,77^ The integration of nanodisc, nanopore, and BLI techniques (ND-BLI) permitted the immobilization of engineered nanopore sensors onto a surface without needing solubilizing detergents for a hydrophobic protein scaffold. This experimental strategy represents the fundamental basis for evaluating engineered nanopore-based sensors in their lipid environment using a confirmatory optical recording in an ensemble. Moreover, this approach creates opportunities for discovering details of single-molecule protein capture kinetics hidden in bulk-phase measurements while using identical nanopore sensors with electrical and optical modalities.

To appraise our attempt at a better understanding of these nanopores, we utilized a recently developed sensing platform^58^ that detects WD40 repeat protein 5 (WDR5),^78−80^ a chromatin-associated hub. The 334-residue highly conserved WDR5 plays an essential role in the regulatory mechanisms of histone 3 lysine 4 (H3K4) mono- and dimethylation.^81−84^ All our engineered nanopore sensors are equipped with a 14-residue WDR5 interaction (Win) motif ligand^60,85^ of mixed-lineage leukemia 4 (MLL4_Win_) (Figure 1b and Table S1 in the Supporting Information). MLL4 is a member of the human MLL/SET1 methyltransferase family.^86^ The MLL4_Win_ ligand forms a complex with WDR5 using a deep-cavity binding pocket, also named the Win binding site (Figure S1 in the Supporting Information).^87,88^ This peptide ligand is covalently attached to the N terminus of tFhuA,^29^ a protein nanopore, through a peptide tether. In single-channel electrical recordings, the nanopore sensor is inserted into a synthetic membrane, and the MLL4_Win_–WDR5 interaction is noted through the modulation in the transmembrane current. To better characterize this WDR5-produced current modulation, we methodically examined targeted variations in the tethering restraint of MLL4_Win_. We also used our proposed ND-BLI platform to better understand the physical process of reversible captures of WDR5 by MLL4_Win_, which occurred outside the nanopore. The resistive-pulse technique revealed which engineered nanopores are sensitive to the presence of WDR5. Remarkably, both protein detection modalities provide similar kinetic landscapes for these functional sensors, despite their radical distinctions in the sampling rate, sensitivity, conceptual formulation, and readout signal. Finally, our experimental strategy using ND-BLI provides a distinctive method for validating and screening single-molecule nanopore sensors.

## Results and Discussion

### Initial Sensor Design to Detect WDR5 via a Tethered Recognition Element

**Figure 2:**
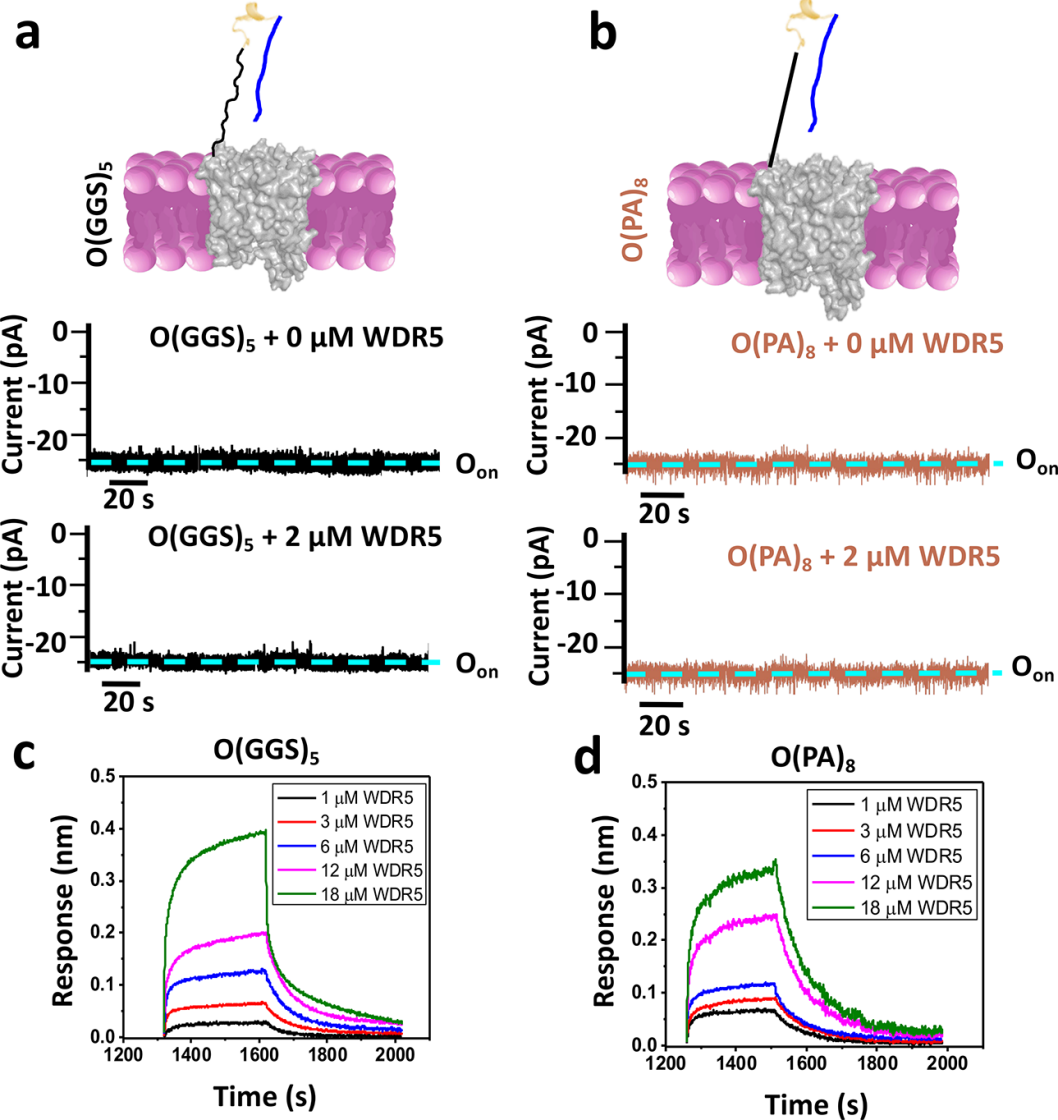
Evaluation of O(GGS)_5_ and O(PA)_8_ nanopore sensors. (a) A nanopore sensor with a peptide adaptor (O) and a flexible (GGS)_5_ tether, also called O(GGS)_5_. Beneath the cartoon are representative single-channel electrical recordings of this nanopore with and without WDR5, showing no signal response (e.g., detection). O_on_ is the WDR5-released substate. These recordings were collected at a transmembrane potential of −20 mV. Here, the signal was low-pass filtered at 100 Hz using an 8-pole Bessel filter. (b) The same as (a) but for O(PA)_8_, which has a rigid (PA)_8_ tether. (c) BLI sensorgrams for O(GGS)_5_ with individual binding curves acquired at different WDR5 concentrations, [WDR5], between 1 and 18 μM (*n* = 5 independent experiments). (d) The same as (c) but for O(PA)_8_.

An advantage of using tFhuA as the nanopore base is its single-polypeptide-chain composition,^89^ which allows straightforward alteration, expression, and refolding in detergents. In addition, tFhuA tolerates large polypeptide extensions at its N terminus without deterioration in pore-forming biophysical properties.^27,29^ Stimulated by previous studies focused on detecting proteins outside the nanopore,^54,56,90^ we decided to attach MLL4_Win_ to tFhuA utilizing a 15-residue flexible tether with the sequence (GGS)_5_. Shorter lengths have also been used, but the complex binding interface between MLL4_Win_ and WDR5,^87,88^ as well as the requirement for a less tethering restraint near the membrane surface, motivated our decision to select a longer linker. Hence, the 15-residue-long tether, which is ∼5.2 nm long in a stretched-out conformation, should provide our recognition element ample space to sample WDR5 in the solution. A 13-residue peptide adaptor (O) was fused to the N terminus of MLL4_Win_, as previously reported.^27,29,58^ Thus, our initial sensor design had a modular structure with an adaptor and a long flexible tether between MLL4_Win_ and tFhuA. For simplicity, we utilized the tether sequence for the nomenclature of all engineered nanopore sensors, making this first sensor O(GGS)_5_ (Table S1 in the Supporting Information). With 300 mM KCl, 20 mM Tris-HCl, 1 mM TCEP, pH 7.5, and a transmembrane potential of −20 mV, this nanopore sensor yielded a quiet open-state current (Figure 2a and Table S2 in the Supporting Information). Surprisingly, adding WDR5 to the *cis* compartment did not change the single-channel electrical signature. Therefore, during the single-molecule evaluation, the O(GGS)_5_ sensor was insensitive to the presence of WDR5.

Next, we asked whether a rigid tether^91−93^ of the same length would produce a different outcome. We decided to replace (GGS)_5_ with a proline-containing (PA)_8_ linker,^91^ which was utilized to create the O(PA)_8_ sensor_._ Again, O(PA)_8_ was insensitive to the presence of WDR5 in the chamber (Figure 2b and Table S2 in the Supporting Information). We hypothesized that both single-polypeptide-chain sensors, O(GGS)_5_ and O(PA)_8,_ adopt conformations that prevent the full exposure of MLL4_Win_ to WDR5. Therefore, the binding interaction was not detectable using the resistive-pulse technique, and it was imperative to test the same modular nanopore sensors using a complementary approach.

Here, we employed ND-BLI for real-time and label-free kinetic measurements between our MLL4_Win_-containing sensors and WDR5 in an ensemble. We utilized membrane scaffold proteins (MSPs),^94^ as well as and buffer conditions similar lipids to those used in single-molecule detection, to form complexes of nanodisc (ND)–nanopore sensors (Experimental Section and Figure S2 in the Supporting Information). The ND–nanopore complexes were immobilized on BLI sensor chips using biotin–streptavidin chemistry. There are two significant advantages of this ND-BLI experimental approach. First, this tactic circumvents the use of detergents. In the absence of NDs, these nanopore studies would require the presence of detergent micelles, increasing the likelihood of protein aggregation and heterogeneity on the surface of the BLI sensor chips. Second, the kinetic measurements of the MLL4_Win_–WDR5 interaction using the ND-BLI optical modality involve an identical nanopore architecture as in single-channel electrical recordings. Moreover, using NDs provides insight into potential interactions between the recognition element and the surrounding lipids. This scenario may prevent the detection of WDR5 by O(GGS)_5_ and O(PA)_8_. If MLL4_Win_ interacted with the lipid membrane, it might not be accessible to bind to WDR5. Therefore, NDs were needed for the reliability of results and more insightful information on what was happening with these sensors.

After reconstitution of the O(GGS)_5_ sensor into an ND, we followed the same strategy to test this sensor via ND-BLI. Surprisingly, O(GGS)_5_ showed concentration-dependent binding with WDR5 (Figure 2c and Figure S3 in the Supporting Information). The association binding curves were acquired by placing the ND-BLI sensors in wells of varying WDR5 concentrations. The dissociation binding curves were collected by placing the ND-BLI sensors in WDR5-free wells. This finding indicates that there was no physical obstacle preventing the MLL4_Win_–WDR5 interaction. Such an optical sensing modality reported an association rate constant, *k*_on_, of (1.9 ± 0.2) × 10^4^ M^–1^ s^–1^ (*n* = 5) and a dissociation rate constant, *k*_off_, of (0.37 ± 0.02) × 10^–2^ s^–1^ (*n* = 5) (Table S3 in the Supporting Information). Furthermore, O(PA)_8_ was tested through ND-BLI and showed the detection of the MLL4_Win_–WDR5 interaction (Figure 2d and Figure S3 in the Supporting Information). In this case, *k*_on_ and *k*_off_ were (1.7 ± 0.1) × 10^4^ M^–1^ s^–1^ and (1.1 ± 0.2) × 10^–2^ s^–1^ (*n* = 5), respectively (Table S3 in the Supporting Information). Further control experiments confirmed that the recorded interactions were between MLL4_Win_ and WDR5. First, NDs without nanopores (empty NDs) showed no interactions with WDR5 (Figure S4a,b in the Supporting Information). Second, no interactions with WDR5 were detected using the ND-reconstituted tFhuA nanopores (Figure S4c,d in the Supporting Information). Although the physical MLL4_Win_–WDR5 interaction occurs in the solution, this is not transduced in a modulated electrical current by either O(GGS)_5_ or O(PA)_8_.

### The Optical Sensing Modality Guided Further Tether Explorations

**Figure 3:**
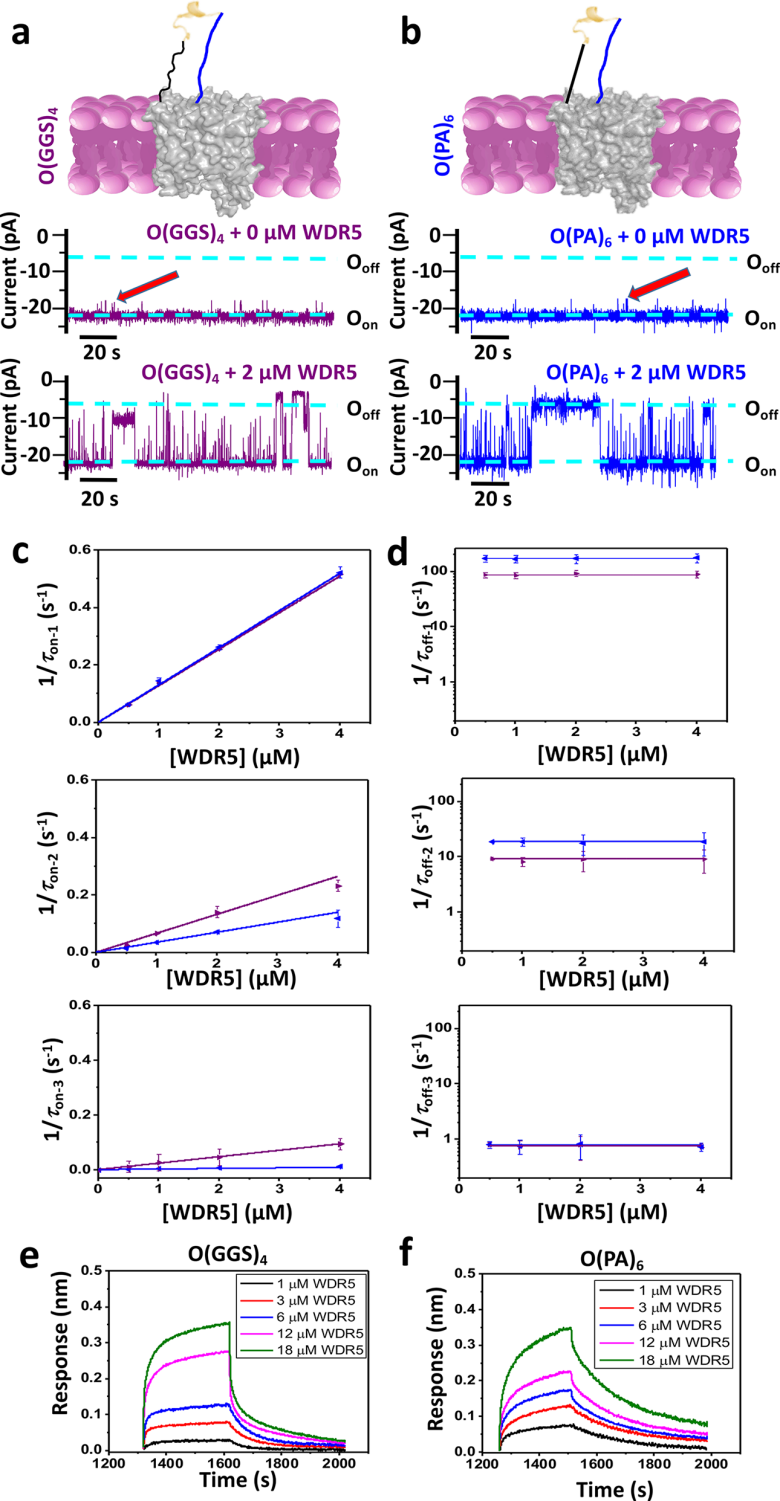
Evaluation of the O(GGS)_4_ and O(PA)_6_ nanopore sensors. (a) A nanopore sensor with a peptide adaptor (O) and a flexible (GGS)_4_ tether, also called O(GGS)_4_. Beneath the cartoon are representative single-channel electrical recordings of this nanopore with and without WDR5. O_on_ and O_off_ are the WDR5-released and WDR5-captured substates, respectively. These recordings were collected at a transmembrane potential of −20 mV. Here, the signal was low-pass filtered at 100 Hz using an 8-pole Bessel filter. (b) The same as (a) but for O(PA)_6_, which has a rigid (PA)_6_ tether. (c) Dose responses of the event frequency, *f*, in the form of 1/τ_on_. For the short-lived, medium-lived, and long-lived binding events, their corresponding event frequencies are 1/τ_on-1_, 1/τ_on-2_, and 1/τ_on-3_, respectively. Values in all panels are mean ± sd for both O(GGS)_4_ (magenta) and O(PA)_6_ (blue) and using a number *n* = 4 and *n* = 3 of independently executed experiments, respectively. (d) Plots indicating dose responses of 1/τ_off-1_, 1/τ_off-2_, and 1/τ_off-3_ for the short-, medium-, and long-lived events, respectively. Values in all panels are mean ± sd for both O(GGS)_4_ (magenta) and O(PA)_6_ (blue) and using a number *n* = 4 and *n* = 3 of independently executed experiments, respectively. (e) BLI sensorgrams for O(GGS)_4_ with individual binding curves acquired at [WDR5] values between 1 and 18 μM (*n* = 3 independent experiments). (f) Same as (e) but for O(PA)_6_ (*n* = 3 independent experiments). In (a) and (b), the red arrows indicate short-amplitude and brief current fluctuations present in the single-channel electrical signature of functional nanopore sensors.

Since O(GGS)_5_ and O(PA)_8_ could detect WDR5 during ND-BLI testing, we postulated that the longer linkers facilitated the MLL4_Win_–WDR5 interaction too far from the pore opening. This way, the peptide adaptor, O, did not reach the pore opening to form nonspecific contacts required for signal modulation.^27,29^ Therefore, we decided to decrease the flexible tether length by one repeat unit (e.g., (GGS)_4_) and create the O(GGS)_4_ sensor. This sensor had an open-state current decorated by low-amplitude and short-lived current spikes (Figure 3a and Table S2 in the Supporting Information). Remarkably, removing one repeat unit from the tether yielded a sensor able to detect WDR5 (Figure 3a). The frequency of binding events, *f*, was amplified by the increase in the WDR5 concentration, [WDR5] (Figure S5 in the Supporting Information). We used the maximum likelihood method^95^ and logarithm likelihood ratio (LLR) tests^96−98^ to determine the probability distribution function (PDF) model of the WDR5-released (τ_on_) and WDR5-captured (τ_off_) durations. We noted a single-exponential probability distribution of WDR5-released events (Figure S6a in the Supporting Information). Interestingly, WDR5-captured durations followed a three-exponential probability distribution, as judged by the LLR values (Figures S6b and S7 in the Supporting Information). For 2 μM WDR5, the probabilities of the short-, medium-, and long-lived events, *P*_1_, *P*_2_, and *P*_3_, were (mean ± sd) 0.58 ± 0.06, 0.28 ± 0.10, and 0.14 ± 0.02, respectively (Table S4 in the Supporting Information). The association rate constants, *k*_on-*i*_ (*i* = 1, 2, and 3), can be calculated as *k*_on-*i*_ = (1/τ_on-*i*_[WDR5]), where τ_on-i_ is the corresponding mean duration of WDR5-released events (Supplementary Tables S5–S6). Here, i = 1, 2, and 3 are subscripts for the short-, medium-, and long-lived current blockades. The dissociation rate constants, *k*_off-*i*_ (*i* = 1, 2, and 3), were determined as the reciprocal of the mean WDR5-captured durations (1/τ_off-i_; *i* = 1, 2, and 3).

Further analysis confirmed that the MLL4win–WDR5 interaction produced the blockades. For example, no current blockades were observed when tFhuA^29^ was exposed to WDR5 added to the *cis* side. This result indicates no nonspecific interaction between tFhuA and WDR5 (Figure S8 in the Supporting Information). Our findings with O(GGS)_4_ prompted the development of a nanopore sensor with a rigid tether of similar size. This nanopore, also named O(PA)_6_, encompassed a (PA)_6_ tether (Figure 3b). Here, we asked whether the three subpopulations of binding MLL4_Win_–WDR5 interactions are still detectable with a rigid tether-containing nanopore. If so, are the kinetics and dynamics of the three-binding events affected by the tether rigidity when its length is altered? We noted that O(PA)_6_ was sensitive to the presence of WDR5. In addition, WDR5-captured durations again followed a three-exponential probability, as assessed by the LLR analysis (Figures S9 and S10 in the Supporting Information). Notably, the probability of the short-lived events, *P*_1_, increased while the probability of the long-lived events, *P*_3_, decreased with the rigid tether compared to the flexible tether. For 2 μM WDR5, the probabilities of the short-, medium-, and long-lived events, *P*_1_, *P*_2_, and *P*_3_, were (mean ± sd) 0.72 ± 0.08, 0.26 ± 0.05, and 0.02 ± 0.01, respectively (Table S7 in the Supporting Information). This finding indicates that the increased restraint via a rigid linker makes it more difficult for MLL4_win_ to sample long-lived binding interactions with WDR5, but it is easier to attain the short-lived events. Using a similar approach, we determined *k*_on-*i*_ and *k*_off-*i*_ (*i* = 1, 2, and 3; Tables S8 and S9 in the Supporting Information). Here, *i* = 1, 2, and 3 are subscripts for the short-, medium-, and long-lived current blockades, respectively.

Alternatively, the association rate constant for these nanopore sensors can be inferred using a linear fit of the event frequency, *f*_*i*_ (*i* = 1, 2, and 3), in terms of 1/τ_on-*i*_ versus [WDR5] (Figure 3c). The association rate constants, *k*_on-*i*_ (*i* = 1, 2, and 3), for O(GGS)_4_ were (1.4 ± 0.2) × 10^5^, (7.9 ± 0.4) × 10^4^, and (4.0 ± 0.5) × 10^4^ M^–1^ s^–1^, respectively (Table S10 in the Supporting Information). The association rate constants, *k*_on-*i*_ (*i* = 1, 2, and 3), for O(PA)_6_ were (1.6 ± 0.2) × 10^5^, (5.1 ± 0.2) × 10^4^, and (0.80 ± 0.04) × 10^4^ M^–1^ s^–1^, respectively. Hence, we observe a decline in the *k*_on-3_ acquired with a rigid (PA)_6_ tether-containing nanopore, likely because of the increased restraint. We can also obtain the dissociation rate constant using a linear fit of 1/τ_off-*i*_ (*i* = 1, 2, and 3) (Figure 3d). For O(GGS)_4_, the dissociation rate constants, *k*_off-*i*_ (*i* = 1, 2, and 3), were 86 ± 3, 9.2 ± 0.5, and 0.78 ± 0.02 s^–1^, respectively (Table S10 in the Supporting Information). For O(PA)_6_, the *k*_off-*i*_ (*i* = 1, 2, and 3) values were 170 ± 8, 18 ± 1, and 0.80 ± 0.02 s^–1^, respectively. We note that the tether rigidity increased the *k*_off-1_ and *k*_off-2_ of the short- and medium-lived events, respectively, which are more sensitive to restraint alterations. On the contrary, we do not see a change in the *k*_off-3_ value of the long-lived events.

We next wanted to test O(GGS)_4_ and O(PA)_6_ using our amalgamated ND-BLI approach for further validation. We anticipated that the ND-BLI results for these 12-residue tether-containing sensors would yield weaker interactions than O(GGS)_5_ and O(PA)_8_ because they have an increased tether restraint. The analysis of O(GGS)_4_ yielded an association rate constant, *k*_on_, of (2.0 ± 0.4) × 10^4^ M^–1^ s^–1^ and a dissociation rate constant, *k*_off_, of (0.39 ± 0.04) × 10^–2^ s^–1^ (*n* = 5; Figure 3e and Figure S11 and Table S11 in the Supporting Information). This finding indicated no significant change in the kinetics of binding interactions between O(GGS)_4_ and O(GGS)_5_ (Tables S3 and S11 in the Supporting Information). In contrast to O(GGS)_5_, O(GGS)_4_ facilitates the transduction of the physical MLL4_Win_–WDR5 interaction outside the pore lumen into an electrical readout during the single-molecule analysis. The *k*_on_ and *k*_off_ values for O(PA)_6_ were (1.1 ± 0.2) × 10^4^ M^–1^ s^–1^ and (1.2 ± 0.1) × 10^–2^ s^–1^, respectively, during ND-BLI measurements (*n* = 5; Figure 3f and Figure S11 and Table S11 in the Supporting Information). Again, decreasing the length of the rigid tether by four residues did not alter the kinetics of the binding events, as revealed by ND-BLI measurements. These findings are in contrast with our predictions for 12-residue tether-containing sensors.

### Validation of Kinetic Landscapes of Single-Molecule and Ensemble Modalities

**Figure 4:**
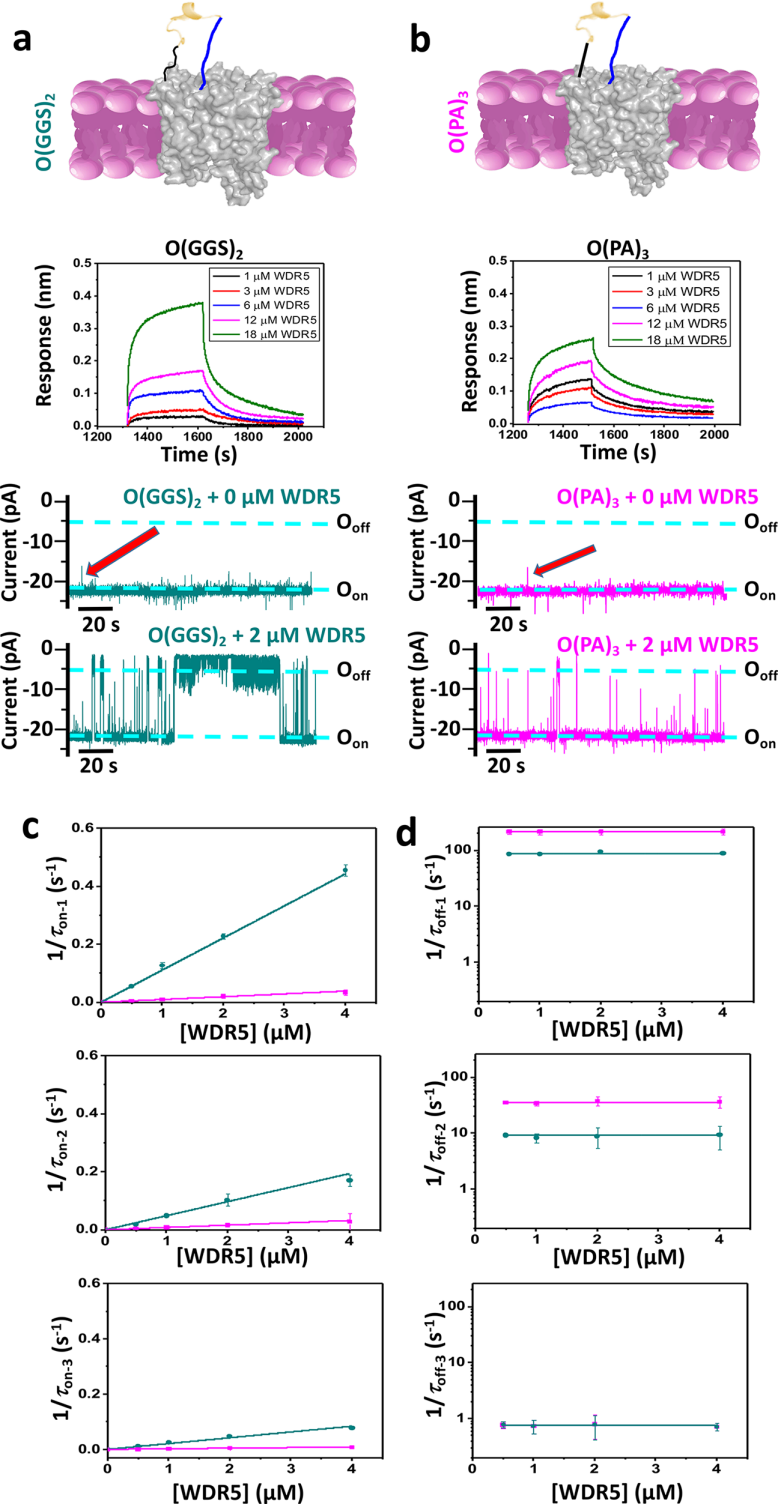
Evaluation of the O(GGS)_2_ and O(PA)_3_ nanopore sensors. (a) A nanopore sensor with a peptide adaptor (O) and a flexible (GGS)_2_ tether, also called O(GGS)_2_. Beneath the cartoon is a diagram with BLI sensorgrams for individual binding curves acquired at [WDR5] values between 1 and 18 μM for O(GGS)_2_ (*n* = 6 independent experiments). This panel also includes representative single-channel electrical recordings of this nanopore with and without WDR5. O_on_ and O_off_ are the WDR5-released and WDR5-captured substates, respectively. These recordings were collected at a transmembrane potential of −20 mV. Here, the signal was low-pass filtered at 100 Hz using an 8-pole Bessel filter. These data were collected using a representative single-channel electrical trace of a reconstituted O(GGS)_2_ nanopore. (b) The same as (a) but for O(PA)_3_, which has a rigid (PA)_3_ tether. (c) Dose responses of the event frequency, *f*, in the form of 1/τ_on_. For the short-lived, medium-lived, and long-lived binding events, their corresponding event frequencies are 1/τ_on-1_, 1/τ_on-2_, and 1/τ_on-3_, respectively. Values in all panels are mean ± sd for both O(GGS)_2_ (cyan) and O(PA)_3_ (magenta) using a number *n* = 6 and *n* = 3 of independently executed experiments, respectively. (d) Plots indicate dose responses of 1/τ_off-1_, 1/τ_off-2_, and 1/τ_off-3_ for the short-, medium-, and long-lived events, respectively. Values in all panels are mean ± sd for both O(GGS)_2_ (cyan) and O(PA)_3_ (magenta) from a number *n* = 6 and *n* = 3 of independently executed experiments, respectively. In (a) and (b), the red arrows indicate short-amplitude and brief current fluctuations present in the single-channel electrical signature of functional nanopore sensors.

Our ND-BLI approach was used to provide evidence that four nanopore sensors (O(GGS)_5_, O(PA)_8_, O(GGS)_4_, and O(PA)_6_) have MLL4_Win_ interacting with WDR5. Because extending the tether length would likely prevent the detection of WDR5 in single-molecule measurements, we decided to evaluate nanopores of shorter tethers. Hence, we pursued the development and comparison of nanopore sensors with a (GGS)_2_ and a (PA)_3_ tether sequence, also named O(GGS)_2_ and O(PA)_3_, respectively. This way, MLL4_Win_ underwent a substantially increased restraint while these tethers permitted an effective separation between the opening of tFhuA and the recognition element. This separation was needed to reduce the likelihood of potentially strong electrostatic interactions between a critical Arg residue^99^ of MLL4_win_ for binding with WDR5 and the negatively charged side chains along the tFhuA opening. Therefore, we expedited the testing of O(GGS)_2_ and O(PA)_3_ via ND-BLI (Figure 4a,b and Figure S12 in the Supporting Information). The *k*_on_ and *k*_off_ values for O(GGS)_2_ were (1.9 ± 0.2) × 10^4^ M^–1^ s^–1^ and (0.46 ± 0.02) × 10^–2^ s^–1^, respectively (*n* = 5; Table S12 in the Supporting Information). For the O(PA)_3_ sensor, the *k*_on_ and *k*_off_ values were (0.60 ± 0.08) × 10^4^ M^–1^ s^–1^ and (1.8 ± 0.2) × 10^–2^ s^–1^, respectively(*n* = 5). These results confirmed that neither *k*_on_ nor *k*_off_ is influenced by the length of the flexible tether. The BLI analysis of rigid tether-containing nanopore sensors showed small changes in the kinetics of binding interactions. However, the ND-BLI-determined *k*_off_ for rigid-linker-containing nanopores is consistently greater than that of flexible ones.

O(GGS)_2_ and O(PA)_3_ were evaluated further via the resistive-pulse technique to determine if these observations are independent of the detection modality. O(GGS)_2_ showed an open-state current and could detect WDR5 (Figure 4a and Table S2 in the Supporting Information). This sensor showed kinetic results like those observed with O(GGS)_4_ (Figures S13 and S14 and Tables S13–S15 in the Supporting Information). The O(PA)_3_ sensor also showed an open-state current and could detect WDR5 through a three-subpopulation distribution of binding events (Figure 4b and Figures S15 and S16 in the Supporting Information). Remarkably, O(PA)_3_ showed a significant decrease in the frequency of events compared to O(PA)_6,_ yet a similar probability of each event type and corresponding dissociate rate constant (Figures 3b and 4b and Tables S8–S10 and S16–S18 in the Supporting Information). For O(GGS)_2_, the association rate constants, *k*_on-*i*_ (*i* = 1, 2, and 3), were (1.1 ± 0.2) × 10^5^, (6.9 ± 0.4) × 10^4^, and (3.5 ± 0.7) × 10^4^ M^–1^ s^–1^, respectively (Figure 4c and Table S19 in the Supporting Information). The corresponding dissociation rate constants, *k*_off-*i*_ (*i* = 1, 2, and 3), were 88 ± 4, 9.4 ± 0.4, and 0.77 ± 0.03 s^–1^, respectively (Figure 4d). For O(PA)_3_, the association rate constants, *k*_on-*i*_ (*i* = 1, 2, and 3), were (0.29 ± 0.09) × 10^5^, (1.0 ± 0.2) × 10^4^, and (0.20 ± 0.04) × 10^4^ M^–1^ s^–1^, respectively. Their corresponding dissociation rate constants, *k*_off-*i*_ (*i* = 1, 2, and 3), were 220 ± 4, 35 ± 1, and 0.79 ± 0.02 s^–1^, respectively. These results determined through the resistive-pulse technique confirm no significant changes in *k*_on-*i*_ and *k*_off-*i*_ among flexible-tether-containing sensors. In contrast, the rigid-linker-containing nanopores show some amplifications in *k*_off-1_ and *k*_off-2_ but a reduction in the *k*_on-*i*_ for a shorter tether length. Finally, the single-molecule analysis shows that flexible tethers produce lower *k*_off-1_ and *k*_off-2_ values than rigid tethers.

### The Coupling of Optical and Electrical Analyses Identifies the Importance of the Adaptor

**Figure 5:**
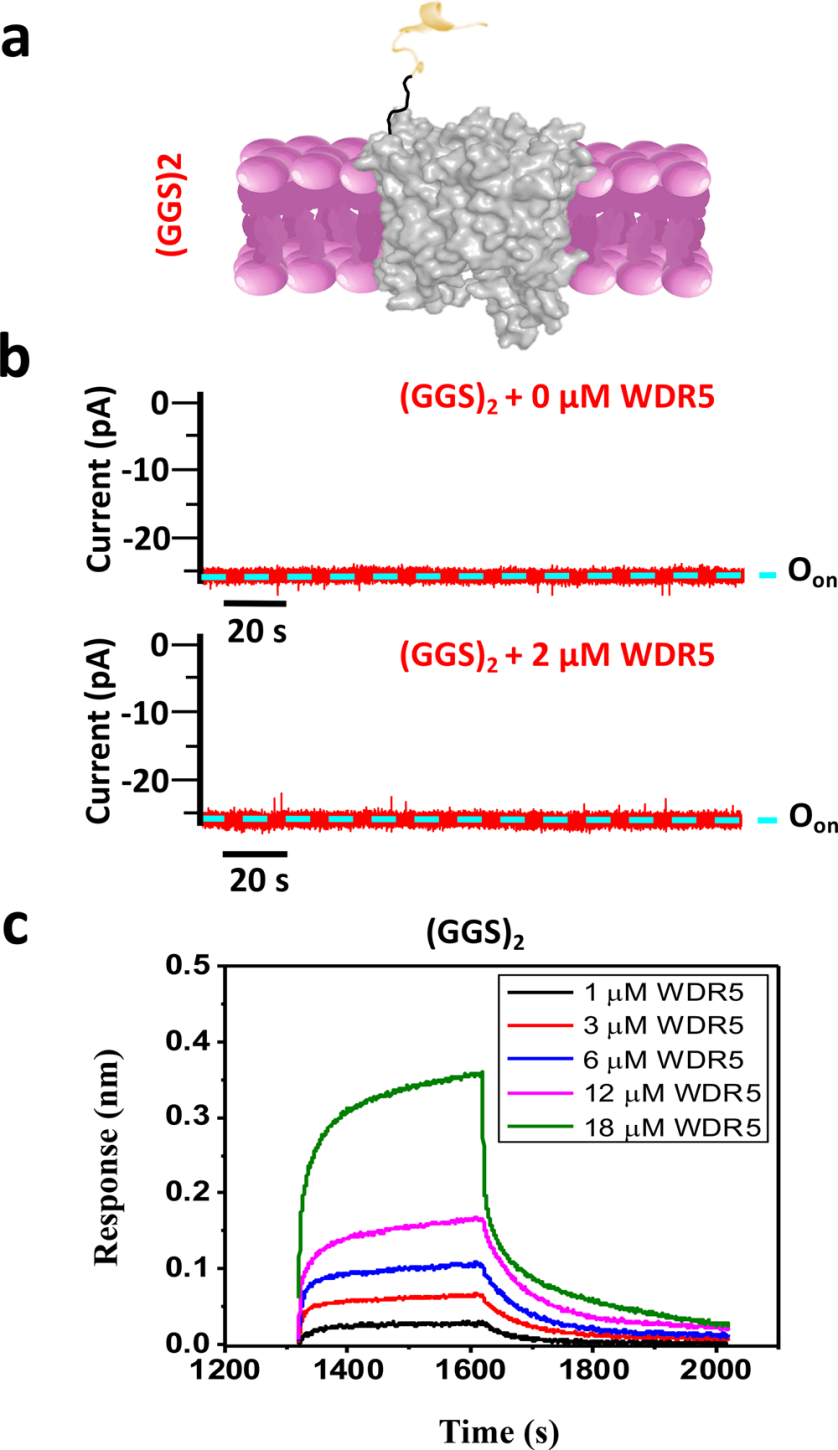
Evaluation of the (GGS)_2_ nanopore sensor. (a) A nanopore sensor with a flexible (GGS)_2_ tether, also named (GGS)_2_. (b) Representative single-channel electrical recordings of this nanopore with and without WDR5. O_on_ is the WDR5-released substate. These single-channel electrical traces indicate no MLL4_Win_–WDR5 interaction (*n* = 4 independently reconstituted nanopores). These recordings were collected at a transmembrane potential of −20 mV. Here, the signal was low-pass filtered at 100 Hz using an 8-pole Bessel filter. (c) BLI sensorgrams with individual binding curves of (GGS)_2_ acquired at [WDR5] values between 1 and 18 μM. These curves show the binding of (GGS)_2_ to WDR5 in a concentration-dependent manner (*n* = 5).

Our dual analysis of these nanopore sensors using electrical and optical analyses showed that ND-BLI could guide their design. Although ND-BLI measurements have a reduced sampling rate, which limits their ability to resolve short-lived and most medium-lived events, they can fill the gaps that single-molecule electrical recordings leave behind. One of these significant gaps is the role of the peptide adaptor. Therefore, we created a nanopore sensor with a (GGS)_2_ tether but without the peptide adaptor, also named (GGS)_2_ (Figure 5a)_._ Since all flexible tethers yielded similar kinetics, we could have used any length, but we selected the 6-residue peptide to ease the data interpretation. Notably, the (GGS)_2_ sensor had a stable open-state current slightly larger than O(GGS)_2_ by ∼4 pA (Figures 4a and 5b and Table S2 in the Supporting Information). In addition, the single-channel electrical signature of (GGS)_2_ is visibly quieter than O(GGS)_2_, lacking low-amplitude flickering fluctuations. Yet, unlike O(GGS)_2_, the (GGS)_2_ sensor could not detect WDR5 (Figure 5b). These findings confirm our aforementioned hypothesis that the adaptor in the functional state undergoes weak nonspecific interactions with the pore opening. The acidic residues of the unstructured adaptor, D3, E7, and E9 (Experimental Section), potentially make electrostatic contacts with basic residues located on the β turns of tFhuA. These residues include R106, K110, K258, R346, R472, and R498. We then tested (GGS)_2_ via ND-BLI. We found that (GGS)_2_ is sensitive to the presence of WDR5 (Figure 5c). The ND-BLI-determined *k*_on_ and *k*_off_ for (GGS)_2_ were (1.9 ± 0.3) × 10^4^ M^–1^ s^–1^ and (0.49 ± 0.03) × 10^–2^ s^–1^, respectively (*n* = 5; Figure S17 and Table S20 in the Supporting Information). It should be noted that sensors O(GGS)_5_ and O(PA)_8_ also exhibited single-channel electrical signatures closely like (GGS)_2_ (Figures 2a,b and 5b and Table S2 in the Supporting Information). This result is in accord with our interpretation that the tethers corresponding to these nanopore sensors are longer than the physical limit of detection. In these cases, the adaptor cannot reach the pore opening. Therefore, we conclude that the lack of nonspecific interactions between the peptide adaptor and tFhuA makes these nanopore sensors insensitive to WDR5 (Table 1). Indeed, the four functional sensors O(GGS)_2_, O(GGS)_4_, O(PA)_3_, and O(PA)_6_ showed reduced unitary current with respect to nonfunctional ones.

**Table 1:** Single-Molecule Electrical Signatures of the Seven Nanopore Sensors Examined in This Work^a^

sensor	type	open-state current	amplified flickering^b^	electrical recordings *K*_D-3_ (μM)	ND-BLI optical *K*_D_ (μM)
**(GGS)**_**2**_	**nonfunctional**^c^	**unchanged**^e^	**no**	**NA**	**0.26 ± 0.03**
O(GGS)_2_	functional^d^	reduced^f^	yes	21 ± 5	0.25 ± 0.05
O(GGS)_4_	functional^d^	reduced^f^	yes	20 ± 3	0.20 ± 0.05
**O(GGS)**_**5**_	**nonfunctional**^c^	**unchanged**^e^	**no**	**NA**	**0.20 ± 0.03**
O(PA)_3_	functional^d^	reduced^f^	yes	718 ± 21	3.01 ± 0.94
O(PA)_6_	functional^d^	reduced^f^	yes	103 ± 11	1.10 ± 0.42
**O(PA)**_**8**_	**nonfunctional**^c^	**unchanged**^e^	**no**	**NA**	**0.62 ± 0.12**

a Rows in boldface correspond to cases where the peptide adaptor interacts with the tFhuA entrance on the *cis* side. All experimental conditions are provided in the Experimental Section.

b Amplified flickering is the occurrence of upward and downward current spikes accompanying the open-state current.

c Nonfunctional nanopore sensors were insensitive to the presence of WDR5 because no WDR5-produced current modulations were detected.

d Functional sensors were utilized to report WDR5-produced current modulations.

e The open-state conductance was not different from that acquired with the adaptor-free nanopore sensor (GGS)_2_.

f The open-state conductance was reduced with respect to that acquired with the adaptor-free nanopore sensor (GGS)_2_.

To better understand whether the adaptor influences the kinetic and equilibrium constants of protein captures, we next compared these parameters obtained for (GGS)_2_ and O(GGS)_2_ using ND-BLI (Table 2). We noted no statistically significant distinctions between these two sensors. This comparison required ND-BLI because this optical platform integrates the same nanopore sensors and surrounding lipids from single-channel electrical recordings. In addition, (GGS)_2_ exhibits ND-BLI-determined kinetics like all other O(GGS)_*n*_ sensors (*n* = 2, 4, 5), clarifying that the peptide adaptor does not influence the results (Table S21 in the Supporting Information). It simply acts as a transducer for the physical MLL4_win_–WDR5 interaction. In the past, a closely related adaptor-induced unitary current reduction was observed with tFhuA to probe protein–protein interactions in a different experimental context.^29^

**Table 2:** Kinetic and Equilibrium Constants for the MLL4_Win_–WDR5 Interaction Using Different Approaches^a^

method	tether/sensor	*k*_on_ (10^–4^ M^–1^ s^–1^)	*k*_off_ (s^–1^)	*K*_D_ (μM)	ref
FP^b^	(GGS)_3_	NA	NA	0.13 ± 0.2	(60)
SPR^b^	(GGS)_3_	21 ± 3	0.041 ± 0.003	0.19 ± 0.02	(60)
BLI^b^	(GGS)_3_	2.3 ± 0.2	0.039 ± 0.002	1.7 ± 0.2	(60)
electrical recordings^c^	(GGS)_2_	NA	NA	NA	this study
electrical recordings^c^	O(GGS)_2_	3.6 ± 0.8	0.78 ± 0.06	21 ± 5	this study
ND-BLI^d^	(GGS)_2_	1.9 ± 0.3	0.0049 ± 0.0003	0.26 ± 0.03	this study
ND-BLI^d^	O(GGS)_2_	1.9 ± 0.2	0.0046 ± 0.0002	0.25 ± 0.05	this study

a Values indicate mean ± s.d. using three independent experiments.

b Steady-state fluorescence polarization (FP) spectroscopy was conducted using sulforhodamine-labeled MLL4_Win_ and WDR5 free in a solution containing 20 mM Tris– HCl, 150 mM NaCl, 1 mM TCEP, 0.005% Tween 20, pH 7.5. Surface plasmon resonance (SPR) experiments were performed using WDR5 immobilized on the sensor surface and unrestrained MLL4_Win_. In this case, a running buffer contained 20 mM Tris-HCl (pH 7.5), 150 mM NaCl, 1 mM TCEP, 0.05% Tween 20. BLI experiments were carried out using MLL4_Win_ immobilized on the sensor surface and unrestrained WDR5. In this case, the buffer was 150 mM NaCl, 20 mM Tris–HCl, 1 mM TCEP, 1 mg/mL bovine serum albumin (BSA), pH 7.5.

c These experiments were performed in a buffer solution containing 300 mM KCl, 20 mM Tris-HCl, 1 mM TCEP, pH 7.5.

d These studies were executed in 300 mM KCl, 20 mM Tris-HCl, 1 mg/mL bovine serum albumin (BSA), 1 mM TCEP, pH 7.5.

**Figure 6:**
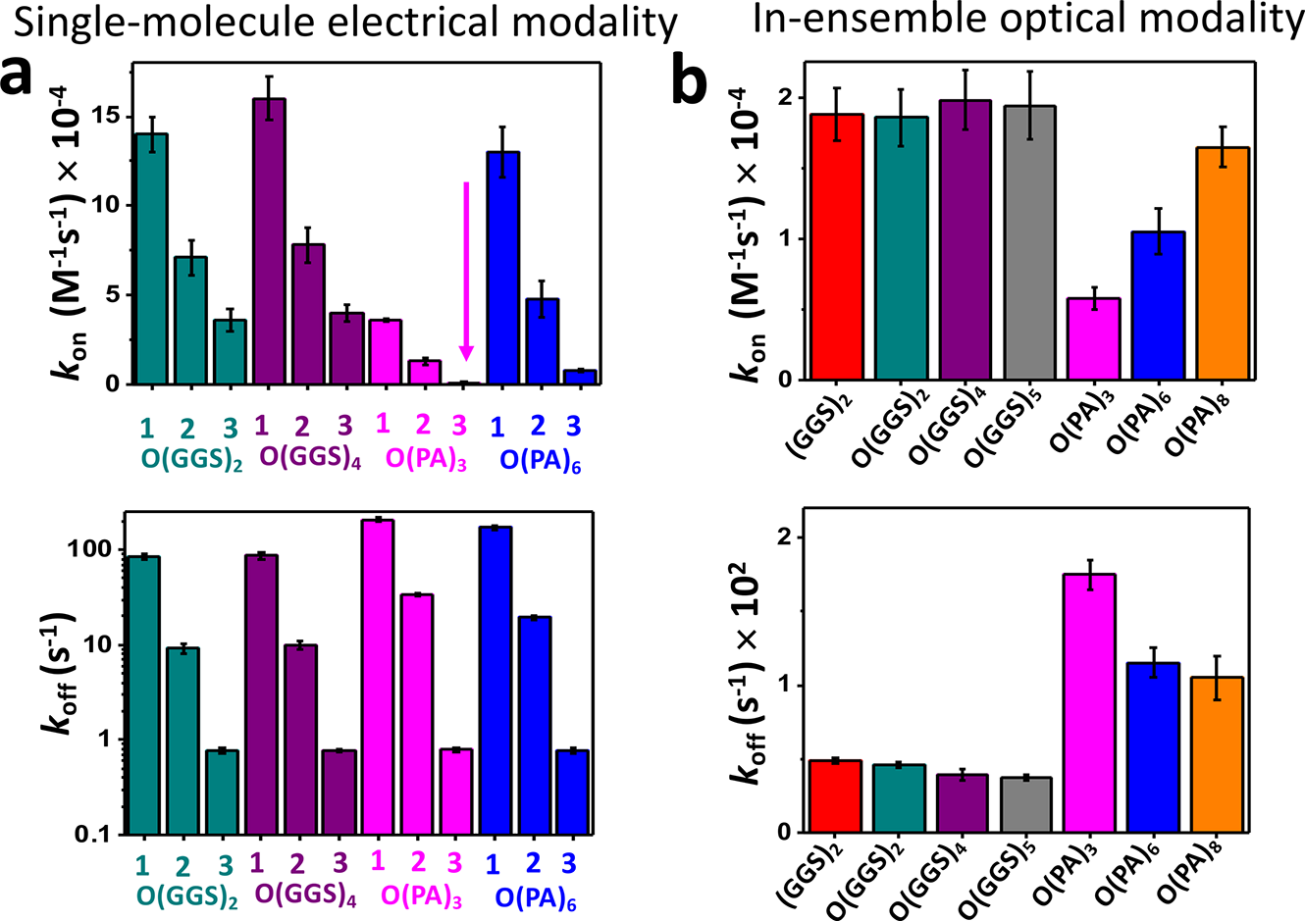
Rate constants of WDR5–MLL4_Win_ interactions using various nanopore sensors amenable to electrical and optical detection modalities. (a) Histograms comparing the association (the top panel) and dissociation (the bottom panel) rate constants determined with various functional single-molecule nanopore sensors. Here, the subscripts 1, 2, and 3 correspond to the short-, medium-, and long-lived binding events, respectively. The arrow in magenta points out the very low value of *k*_on-3_ for O(PA)_3_. (b) Histograms comparing the association (*k*_on_; the top panel) and dissociation (*k*_off_; the bottom panel) rate constants determined by BLI. These experiments were conducted using seven nanopore sensors amenable to an optical protein detection modality.

The coupling of electrical recordings and ND-BLI screening in an ensemble of sensors revealed the power of single-molecule sensing, pointing out the multimodal recognition of WDR5 in the form of a three-subpopulation distribution of binding events. These three binding events correspond to various configurations that the flexible MLL4_Win_ peptide ligand can take when interacting with the deep binding cavity of the Win site of WDR5.^87^ These kinetic details are typically hidden in in-ensemble measurements,^100^ such as those made through ND-BLI. In addition, this study illuminated that the alteration in tether parameters can quantitatively influence the nanopore sensor performance. The ND-BLI application superseded the electrical recording in showing the pattern between results and tether properties, since a tether length longer than 12 residues yielded nonfunctional sensors in the single-molecule analysis (Figure 6). The amplification in *k*_on_ for the longer-rigid tether-containing sensor is in accord with the fly-casting mechanism of association between surface-immobilized recognition elements and their targeted proteins.^61,101,102^ This outcome also agrees with a greater separation between the domains fused through rigid linkers than for the flexible ones.^91^ The increase in *k*_off-1_ and *k*_off-2_ for a short rigid-tether-containing sensor (e.g., O(PA)_3_) likely results from an interfacial repulsion due to volume-exclusion effects,^103^ pulling WDR5 away from the surface. In contrast, *k*_off-3_, which corresponds to the long-lived binding events, is not affected by these repulsion forces, probably because of a relatively more robust MLL4_Win_–WDR5 interaction in this case. The probability of long-lived events with a flexible tether is around 14%, dropping to only 2% with a rigid tether (Table S22 in the Supporting Information). Hence, the rigid tether makes it more difficult for MLL4_Win_ to achieve the conformation required for the long-lived event,^104^ yet it does not influence the most robust interaction because we do not see a change in the *k*_off-3_ (Tables S23 and S24 in the Supporting Information).

The evaluation of these sensors using both approaches show that O(GGS)_2_ and O(GGS)_4_ yielded the strongest WDR5–MLL4_Win_ interaction (Table S25 in the Supporting Information). Hence, they represent the optimized sensor design for our single-molecule electrical recordings. Notably, O(PA)_6_ probes a 5-fold weaker interaction than the optimal sensor design. O(PA)_3_ yields the weakest interaction among all sensors by at least 1 order of magnitude. Therefore, we show that sensors with flexible tethers were more resilient to modifications of the MLL4_Win_ attachment and sampled optimized MLL4_Win_–WDR5 binding. Comparisons of electrical and optical modalities also demonstrate that our design requires the recognition element to be within a physical detection limit with respect to the pore opening.

Although all experiments in this study involve micromolar concentrations of WDR5, the detection threshold using the resistive-pulse technique and nanopores can routinely reach nanomolar^28,29,55−57^ and even picomolar^105^ levels of proteins. On one hand, the detection threshold can be improved through amplification of the capture rate of proteins via electrostatic interactions (e.g., lowering the salt concentration), driving force (e.g., increasing the transmembrane potential), or local electroosmotic pressure (e.g., enriching the sample buffer with osmolytes). On the other hand, the sensitivity of protein nanopore detectors can be enhanced via a significant decrease in the dissociation rate constant through strong-affinity protein analyte-tethered ligand interactions. For example, Zhang and colleagues^59^ have recently utilized multivalent interactions mediated by a nanobody-functionalized nanopore to detect protein biomarkers at picomolar concentrations. In addition, ND-BLI can also be used to probe nanomolar levels of proteins. In this case, the expected detection sensitivity can reach 0.01 × *K*_D_, where *K*_D_ is the equilibrium dissociation constant between the protein analyte and the ligand immobilized on the BLI sensor surface.

## Conclusions

These nanopore-based sensors have been broadly utilized for label-free and real-time protein detection over the last couple of decades. While they are powerful sensing elements, their need for extensive engineering and tedious screening routes is undeniable, limiting their immediate applications. Every time one wants to detect a different protein, there need to be multiple constructs created and screened to validate the optimal sensor design adequately. While there is still a need for heavy protein design, our work provides a platform for an additional screening and validation path. Using ND-BLI drastically reduces the time needed to optimize these sensors. One can determine the kinetics between a target protein and two different constructs in only 30 min using a simple ND-BLI protocol. Also, optical protein detection can fill fundamental gaps when a sensor cannot recognize a targeted protein analyte during the single-molecule analysis. For example, this experimental strategy can be applied to challenging situations when a small protein,^28,29,59^ which serves as a recognition element, must be fused to a nanopore. Suppose the nanopore sensor is insensitive to the presence of a protein analyte. In that case, there are two possibilities: (i) the target protein binds to the recognition element, but a current response is not detectable; (ii) the binding interface of the recognition element is not fully accessible, preventing its specific interaction with the target protein. The ND-BLI sensing approach can provide insight into which possibility is actual. Here, we emphasize that the orientation of the recognition element with respect to the pore opening and the geometry of its complex with the protein analyte play a pivotal role in the overall sensor design. For example, the adaptor is sometimes unnecessary for transducing the protein captures into an electrical readout.^28,33,56^ In this study, we clarify that protein detection cannot be achieved without an adaptor peptide. Furthermore, using ND-BLI facilitated the outcome that weak nonspecific interactions between the adaptor and tFhuA do not impact the real-time kinetics. We also show how a certain restraint on a tethered recognition element affects the kinetics and dynamics of protein recognition using a single-molecule setting. Therefore, our work can act as a roadmap for how others designing nanopore-based sensors can better understand the diverse aspects of their nanopore architectures, compositions, and functions at faster rates.

In summary, we employed membrane protein engineering, nanopore, and nanodisc technologies to develop sensors amenable to single-molecule and bulk-phase protein detection modalities. Both techniques involve real-time and label-free measurements where sensors are immobilized onto a surface and probe a target protein free in solution. Noteworthy, the optical detection modality uses nanodiscs to remove the need for detergents and provide identical lipid surroundings to the resistive-pulse technique. Furthermore, we show that the ND-BLI measurements replicated the effects of the tether length and flexibility observed using single-molecule electrical modality. This finding reinforces that it can provide additional insight into how each sensor will perform and generate a reliable screening approach.

## Experimental Section

### Modular Genetic Engineering

The *omll4(ggs)*_*2*_*tfhua* gene was obtained from GenScript (Piscataway, NJ). From the N to C terminus, this gene encoded a 13-residue adaptor peptide (O, MGDRGPEFELGTM), a 14-residue mixed lineage leukemia 4 (MLL4) Win motif peptide ligand (MLL4_Win_, LNPHGAARAEVYLR), a Gly/Ser-rich tether ((GGS)_2_), and a 455-residue truncation variant of *Ferric hydroxamate uptake component A* of *Escherichia coli* (tFhuA).^29^ All other constructs were created using the site-directed mutagenesis kit from New England Biolabs (Ipswich, MA). For all constructs, pPR-IBA1-*omll4(ggs)_2_tfhua* was utilized as the template. The first modifications included the insertion of additional 6-residue and 9-residue Gly/Ser-rich sequences to create *omll4(ggs)*_*4*_*tfhua* and *omll4(ggs)*_*5*_*tfhua*, respectively. Then, we deleted the adaptor peptide from the original sequence to form *mll4(ggs)*_*2*_*tfhua*. The tether was then substituted with a 6-residue Pro/Ala-rich sequence to generate *omll4(pa)*_*3*_*tfhua*. 6-residue and 10-residue Pro/Ala-rich sequences were also added to *omll4(pa)*_*3*_*tfhua* to develop *omll4(pa)*_*6*_*tfhua* and *omll4(pa)*_*8*_*tfhua*, respectively. The MLL4_Win_ in all constructs represented a recognition element for the targeted protein analyte WDR5 (see below). The adaptor peptide was unstructured in solution.^106^

### Expression and Purification of Protein Nanopores

The MLL4_Win_tFhuA constructs were expressed and purified, as previously described.^27,89^ In brief, cells were induced with 1 mM isopropyl-β-d-1-thiogalactopyranoside (IPTG), harvested, and resuspended in 300 mM KCl, 20 mM Tris-HCl, 5 mM ethylenediaminetetraacetic acid (EDTA), pH 8.0. Cells were lysed with a microfluidizer (Model 110L; Microfluidics, Newton, MA), and the cellular pellets went through a series of Triton washes. Finally, the supernatant was pelleted and solubilized in 8 M urea before being purified on an anion-exchange column (Q12-Sepharose; Bio-Rad, Hercules, CA). For further purification, the samples were passed through a size-exclusion column (HiLoad 16/600 Superdex-75; GE Healthcare Life Sciences, Pittsburgh, PA) and lyophilized.

### Expression and Purification of WDR5

The protein analyte, WDR5_ΔN_,^104^ a truncation WDR5 mutant lacking the residues 1–22, was expressed and purified as previously described.^87,99,104^ The WDR5-containing supernatant underwent initial purification via a metal-affinity column (5 mL, Bio-Scale Mini Profinity IMAC cartridge; Bio-Rad, Hercules, CA). Then two enzymatic assays were performed on the sample. Tobacco Etch Virus (TEV) protease (New England Biolabs) removed the hexahistidine tag, and the benzonase nuclease (Sigma-Aldrich, St. Louis, MO) digested DNA contaminants. Finally, the sample was again passed through the metal-affinity column, and a 10 kDa molecular weight concentrator (Millipore Sigma, St. Louis, MO) was used to concentrate the final protein samples.

### Functional Reconstitution of Protein Nanopore Sensors in Detergents

All MLL4_Win_tFhuA proteins were refolded in *n*-dodecyl-β-d-maltopyranoside (DDM; Anatrace, Maumee, OH) as previously described.^19,28^ After 72 h of dialysis in 200 mM KCl, 20 mM Tris-HCl, pH 8 at 4 °C, the refolded proteins were centrifuged to remove unfolded precipitates.

### Expression and Purification of Membrane Scaffold Protein

The plasmid for the expression and purification of the membrane scaffold protein (MSP) was based on the pMSP1E3D1 plasmid with an N-terminal extension of 7 × Histidine tag, biotin-acceptor peptide purification tag, and a Precision protease cleavage site.^107^*E. coli* BL21(DE3) cells were used to transform the MSP gene-containing plasmid, and a small starter culture was grown in a Luria–Bertani medium with 0.2% glucose overnight at 37 °C. The small culture was transferred to 4–6 L and grown at 37 °C until OD_600_ reached a value of ∼0.55. After initial growth, the culture was induced with 0.5 mM IPTG for 4 h at 37 °C. The cells were centrifuged at 3000*g* for 25 min at 4 °C. The pellet was resuspended in 250 mM KCl, 20 mM Tris-HCl, 10 mM imidazole, pH 8.0. The resuspension was spun at 3000*g* for 15 min, and the supernatant was discarded. The sample was then prepared for lysis by resuspension in 250 mM KCl, 20 mM Tris-HCl, 10 mM imidazole, 8 M urea, pH 8.0. The sample was sonicated for 40 s with two 20 s intervals to shear the genomic DNA. The broken cells were centrifuged at 15000*g* for 20 min at room temperature to separate insoluble from soluble components. The supernatant was passed over a pre-equilibrated NiNTA column for refolding and purification (5 mL, Bio-Scale Mini Profinity IMAC cartridge; Bio-Rad). The elution fractions were collected and run on an SDS-PAGE gel to identify the purity and size of the 7 × histidine-tagged MSP. Biotinylation of MSP was executed on NiNTA beads.

### Expression and Purification of the BirA Enzyme

The plasmid encoding BirA carrying a C-terminal 6 × Histidine tag (pET21a-BirA) was a gift from Alice Ting (Addgene plasmid # 20857; RRID:Addgene_20857). BirA was expressed in BL21(DE3) cells grown in LB containing 0.2% glucose and supplemented with 100 μg/mL carbenicillin. Expression was induced overnight at OD_600_ ≈ 0.6 with 0.5 mM IPTG at 20 °C, followed by centrifugation, resuspension in Buffer A (20 mM Tris, 250 mM NaCl, 10 mM imidazole, pH 8), and freezing at −20 °C until use. Cells were supplemented with Lysozyme (1 mg/mL) and DNaseI (0.1 mg/mL) and incubated on ice for 30 min. Sonication was used to lyse cells in 1 mM PMSF, and the lysate was cleared by centrifugation at 13000*g* for 40 min at 4 °C. Cleared lysate was filtered through a 0.45 um filter and applied to a 10 mL NiNTA column attached to an FPLC at 1 mL/min. Once the sample was loaded, 5 CV of Buffer A was used to wash the column, and BirA was eluted in a linear imidazole gradient with 0–60% Buffer B (20 mM Tris, 250 mM NaCl, 500 mM Imidazole, pH 8). The fractions were resolved on an SDS-PAGE gel, and proper fractions were concentrated by ultrafiltration and applied to a Superdex 75 column, which was attached to an FPLC in 20 mM Tris, 100 mM NaCl, pH 7. The yield from 1 L of cells was ∼25 mg.

### Biotinylation of MSP via BirA

The enzyme BirA was mixed with MSP at a ratio of 1:100 in a buffer containing 150 μM biotin, 5 mM ATP, and 4 mM MgCl_2_, pH 8.0 for biotinylation.^108^ The mixture was placed in a horizontally rotating gravity column. It was left to rotate for 4 h at room temperature. After mixing, the gravity column was opened and washed with 250 mM KCl, 20 mM Tris-HCl, 10 mM imidazole, pH 8.0. Then the biotinylated MSP was eluted by running 250 mM KCl, 20 mM Tris-HCl, 500 mM imidazole, pH 8.0. To test the efficiency of biotinylation, streptavidin beads were used as a pull-down assay to separate reacted from unreacted MSP. An SDS-PAGE gel was utilized to confirm the purity and size of the protein.

### Functional Reconstitution of Protein Nanopore Sensors in Nanodiscs

The nanodisc (ND) fabrication and MLL4_Win_tFhuA reconstitutions occurred in one step. This step began by mixing detergent-solubilized MLL4_Win_tFhuA constructs at a 2:1 MSP:MLL4_Win_tFhuA ratio. The detergent concentration was kept at 1% *n*-dodecyl-β-d-maltopyranoside (DDM; Anatrace). Also, 1,2-diphytanoyl-*sn*-glycero-phosphatidylcholine lipids (Avanti Polar Lipids, Alabaster, AL) were added to the mix at a 1:2:4 MLL4_Win_tFhuA:MSP:lipid ratio. This solution was left to mix at 4 °C for 1 h. Then, 0.4 g/mL of activated Bio-Beads (Gold Biotechnology, Olivette, MO) was added to remove the detergent. The Bio-Bead mixture was rotated at 4 °C for 2 h. Bio-Beads were separated from the supernatant through centrifugation at 4 °C for 5 min at 5000*g*. The sample was run on a size-exclusion column for final purification to collect the elution peaks with the nanodisc-reconstituted MLL4_Win_tFhuA constructs.

### Biolayer Interferometry

Biolayer interferometry (BLI) data collection was performed utilizing an Octet Red384 instrument (FortéBio, Fremont, CA).^60^ Streptavidin (SAX) sensors were soaked in 300 mM KCl, 20 mM Tris-HCl, 1 mg/mL bovine serum albumin (BSA), 1 mM TCEP, pH 7.5 for 30 min. A flexible 31-residue peptide spacer was present between the nanodisc and SAX sensor. 15 nM biotinylated nanodiscs with reconstituted nanopores were loaded onto the sensors for 15 min. Washing off the unbound nanodiscs was achieved by dipping the sensors into a nanodisc-free buffer for 5 min. A serial dilution of WDR5 ranging from 1 to 18 μM was conducted to explore the association phase. Then, the BLI sensors were soaked in a WDR5-free buffer solution to inspect the dissociation process. For all WDR5 concentrations, the empty nanodiscs (e.g., nanodiscs without MLL4_Win_tFhuA) were run as control and accounted for drift. All BLI experiments were performed at 24 °C. The FortéBio Octet Data Analysis software package (FortéBio) was used for data processing and analysis. The association and dissociation rate constants were inferred, as previously reported.^60^

### Single-Molecule Electrophysiology

Single-channel electrical recordings using planar lipid bilayers were conducted, as previously described.^98,109^ Lipid bilayers were made of 1,2-diphytanoyl-*sn*-glycero-phosphatidylcholine (Avanti Polar Lipids) across a 90 μm diameter aperture in the Teflon partition separating the halves of the chamber. The buffer solution was 300 mM KCl, 20 mM Tris-HCl, 1 mM TCEP, pH 7.5. Protein samples of nanopores and analytes were added to the *cis* side at the ground. The electrical signals were acquired using an Axopatch 200B patch-clamp amplifier (Axon Instruments, Foster City, CA). The transmembrane applied potential was −20 mV. The signal was low-pass filtered using an 8-pole Bessel filter (Model 900; Frequency Devices, Ottawa, IL) at a frequency of 10 kHz. A low-noise acquisition system (Model Digidata 1440A; Axon Instruments) was employed to digitize the collected data. The sampling frequency was 50 kHz. 20 min long single-channel electrical traces were additionally filtered at a frequency of 1 kHz for the analysis of binding events. All electrical recordings were performed at room temperature (23 ± 1 °C). Measurements were also made at a frequency of 10 kHz using an Orbit 16 multichannel platform (Nanion Technologies, Inc., Munich, Germany).

### Statistical Analysis of Single-Molecule Events

ClampFit 10.7 (Axon Instruments) and Origin 8.5 (OriginLab, Northampton, MA) were employed to prepare figures. pClamp 10.5 (Axon) was employed for data acquisition and analysis. The maximum likelihood method (MLM)^95^ and logarithm likelihood ratio (LLR)^96−98^ tests were used to fit event duration histograms and compare the results from different statistical models. These approaches were employed to determine the number of statistically significant subpopulations best represented in the data. For example, the best model for the WDR5 release durations was a single-exponential distribution at a confidence number of 0.95. In contrast, the best model for the WDR5 capture durations was a three-exponential distribution.

### Molecular Graphics

All cartoons showing molecular graphics were prepared using the PyMOL Molecular Graphics System (Version 2.4.0; Schrödinger, LLC). In this study, we utilized entries 1BY3 (FhuA) and 4ERZ (WDR5) from the Protein Data Bank for molecular visualizations.

## Supporting Information Available

Peptide sequences for all inspected nanopores, open-state currents of various nanopore sensors, development of the ND-BLI method, fits of the ND-BLI sensorgrams for all inspected nanopores, ND-BLI-determined kinetic rate constants of association and dissociation for all inspected nanopores, ND-BLI sensorgrams of negative- and positive-control experiments, single-channel traces for all functional nanopores at various WDR5 concentrations, duration histograms of the WDR5-released and WDR5-captured events for all functional nanopores at various WDR5 concentrations, kinetic and affinity constants for all functional nanopores and quantitative comparisons between the resistive-pulse technique and ND-BLI (PDF)
