# Supplementary material for: Evaluation of Nanopore Sensor Design Using Electrical and Optical Analyses
Source: ACS Nano. 2023 Jun 1;17(11):10857–71. doi: 10.1021/acsnano.3c02532 (PMC10278182; doi:10.1021/acsnano.3c02532)
Supplement: Supplementary file 1 — nn3c02532_si_001.pdf [file nn3c02532_si_001.pdf]

SUPPORTING INFORMATION FILE

Evaluation of Nanopore Sensor Design Using Electrical and Optical Analyses

Lauren A. Mayse[1,2], Ali Imran[1], Yazheng Wang[1,2], Mohammad Ahmad[1], Rebecca A. Oot[3], Stephan Wilkens[3], and Liviu Movileanu[1,2,4,&]

[1]*Department of Physics, Syracuse University, 201 Physics Building, Syracuse, New York 13244-1130, USA*

[2]*Department of Biomedical and Chemical Engineering, Syracuse University, 329 Link Hall, Syracuse, New York 13244, USA*

[3]*Department of Biochemistry and Molecular Biology, State University of New York - Upstate Medical University, 4249 Weiskotten Hall, 766 Irving Avenue, Syracuse, New York 13210, USA*

[4]*The BioInspired Institute, Syracuse University, Syracuse, New York 13244, USA*

[&]The corresponding author's contact information:
Liviu Movileanu, PhD, Department of Physics, Syracuse University, 201 Physics Building, Syracuse, New York 13244-1130, USA. Phone: 315-443-8078; E-mail: lmovilea@syr.edu

Supplementary Table S1. Peptide sequences for all inspected nanopores. All seven nanopore constructs had the same recognition element. This is the 14-residue Win motif ligand of mixed lineage leukemia 4 (MLL4$_{Win}$; marked in black),[1-4] a histone 3 lysine 4 (H3K4) methyltransferase. The sequence of MLL4$_{Win}$ is LNPHGAARAEVYLR. All sensors maintain the tFhuA nanopore stem (marked in blue).[5, 6] A negative-control nanopore sensor, (GGS)$_2$, does not include a peptide anchor, O (marked in green). The next three sensors have varying lengths of a flexible (GGS)-based tethering arm (marked in red).[7-10] The last three constructs consist of a rigid (PA)-based tethering arm (marked in red) with varying lengths.[7-10]

Sensor nomenclature	Tether sequence	Sequence of the nanopore sensor
(GGS)$_2$	(GGS)$_2$	MLNPHGAARAEVYLRGGSGGStFhuA
O(GGS)$_2$	(GGS)$_2$	MGDRGPEFELGTMLNPHGAARAEVYLRGGSGGStFhuA
O(GGS)$_4$	(GGS)$_4$	MGDRGPEFELGTMLNPHGAARAEVYLRGGSGGSGGSGGStFhuA
O(GGS)$_5$	(GGS)$_5$	MGDRGPEFELGTMLNPHGAARAEVYLRGGSGGSGGSGGSGGStFhuA
O(PA)$_3$	(PA)$_3$	MGDRGPEFELGTMLNPHGAARAEVYLRPAPAPAtFhuA
O(PA)$_6$	(PA)$_6$	MGDRGPEFELGTMLNPHGAARAEVYLRPAPAPAPAPAPAtFhuA
O(PA)$_8$	(PA)$_8$	MGDRGPEFELGTMLNPHGAARAEVYLRPAPAPAPAPAPAPAPAtFhuA

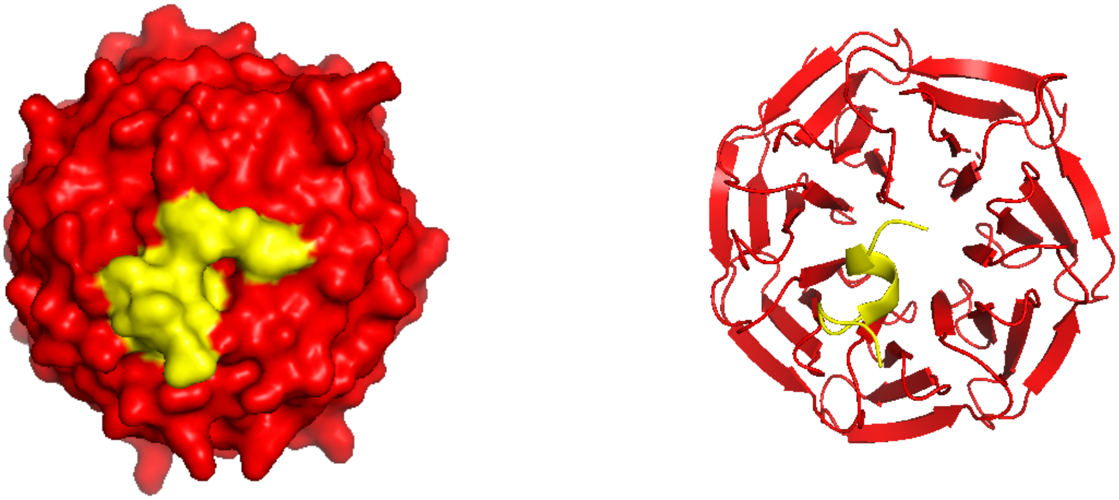

Supplementary Figure S1. Top-view cartoons of the WDR5 interacting with MLL4$_{Win}$ via a deep WDR5 cavity. The WDR5 cavity is also named the Win binding site. The left-side cartoon shows a surface representation of MLL4$_{Win}$ (marked in yellow)[1-4] interacting with WDR5 (marked in red).[11, 12] The right-side cartoon presents the 3_{10}-helix conformation of the MLL4$_{Win}$ ligand (marked in yellow) interacting with WDR5 (marked in red) cavity.[13]

Supplementary Table S2. The open-state currents of various nanopore sensors. The open-state current, I_O, is directly measured at a transmembrane potential of -20 mV. The normalized open-state current, I_N, is the open-state current of the respective nanopore sensor divided by the open-state current of the unmodified tFhuA, which is -30 ± 3 pA (n= 9 independently reconstituted nanopores).

**Nanopore Sensor**	I_O **(pA)**	I_N **(pA)**
**O(GGS)$_5$**	-26 ± 2	0.9 ± 0.4
**O(PA)$_8$**	-25 ± 1	0.8 ± 0.2
**O(GGS)$_4$**	-23 ± 2	0.8 ± 0.3
**O(PA)$_6$**	-22 ± 2	0.7 ± 0.3
**O(GGS)$_2$**	-22 ± 1	0.7 ± 0.1
**O(PA)$_3$**	-23 ± 1	0.8 ± 0.2
**(GGS)$_2$**	-26 ± 3	0.9 ± 0.3

Values are mean ± s.d. Data was obtained using *n* = 3, 3, 4, 3, 6, 3, and 4 independently reconstituted nanopores for O(GGS)$_5$, O(PA)$_8$, O(GGS)$_4$, O(PA)$_6$, O(GGS)$_2$, O(PA)$_3$, and (GGS)$_2$, respectively. The other experimental conditions were the same as those stated in **Experimental Section**.

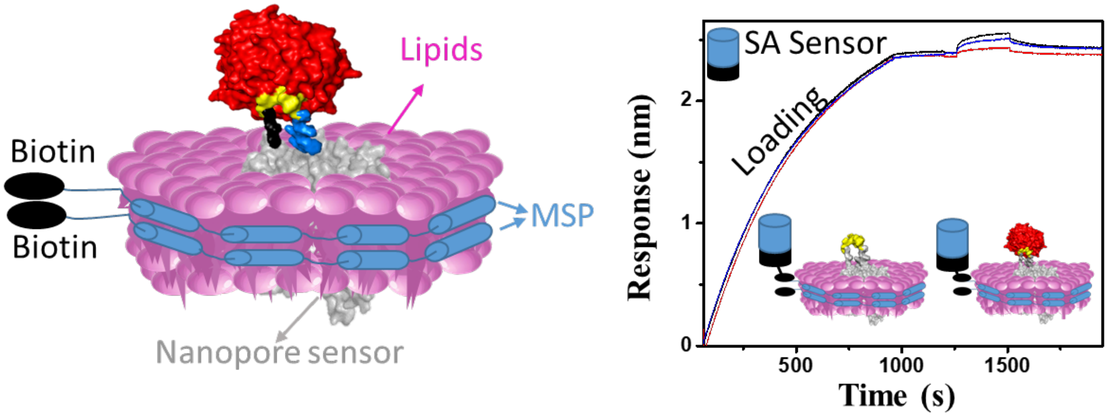

Supplementary Figure S2. Development of the ND-BLI method. (a) A cartoon of a nanopore sensor with a tether (black), recognition element (yellow) and adaptor peptide(blue). This nanopore sensor detect WDR5(red) and has been reconstituted into a biotinylated MSP-mediated ND. Two biotinylated (black) MSP (light blue tubes) dimerize and fill with lipids (magenta) to form a nanodisc (ND). (**b**)An ND-BLI sensorgram showing the loading of the ND-nanopore system onto the streptavidin (SA)-based sensors and their interaction with WDR5. The loading was conducted from 0 to ~950 s, and subsequent only buffer (~900 – 1250 s) as well as WDR5 binding (1250-1450 s) and release (1450-2000 s) via the O(GGS)$_2$ sensor.

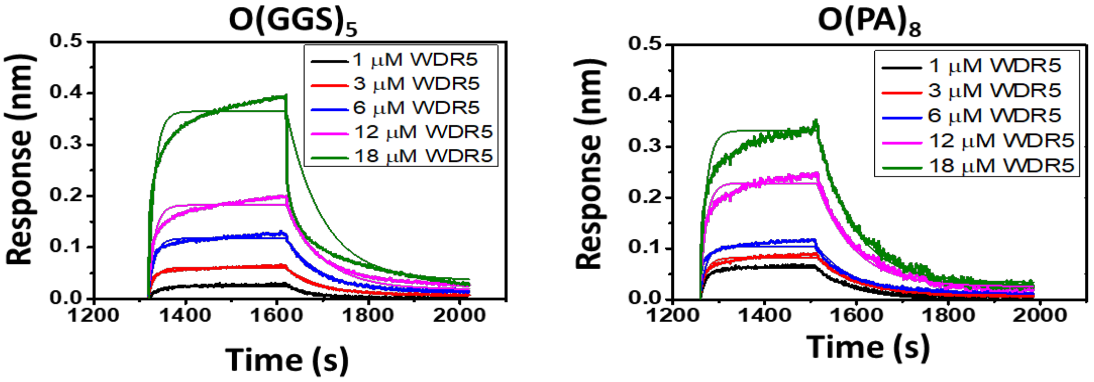

Supplementary Figure S3. Fits of the ND-BLI sensorgrams for O(GGS)$_5$ and O(PA)$_8$. The FortéBio Octet Data Analysis software (FortéBio) was used for the fitting of binding curves. The binding curves are in **Fig. 2c** and **Fig. 2d**, respectively. The curves of the association process were fitted using the following equation: $Y = Y_\infty - (Y_\infty - Y_0)exp(-k_{obs}t)$.[14] Here, Y_0 and Y_∞ are the response signals during the association process at zero and infinity times, respectively. t is the cumulative time of the association reaction. k_{obs} denotes the apparent first-order reaction rate constant of the association process. The dissociation process was fitted using the following equation:
$Y = Y_\infty + (Y_0 - Y_\infty)exp(-k_{off}t)$, where Y_0 and Y_∞ are the response signals during the dissociation process at zero and infinity times, respectively. k_{off} shows the dissociation rate constant. The fits are shown as thin lines with the corresponding color to the curve. Due to the elevated WDR5 concentration, [WDR5], at 12 μM and 18 μM we observe more response (signal) drift, which is the basis for some distinctions between the fits and binding curves.

Supplementary Table S3. ND-BLI-determined kinetic rate constants for O(GGS)$_5$ and O(PA)$_8$. The association and dissociation rate constants were inferred, as previously reported.[1]

Nanopore Sensor	k_{on} ($M^{-1}s^{-1}$) × 10^{-4}	k_{off} (s^{-1}) × 10^{2}
**O(GGS)$_5$**	1.9 ± 0.2	0.37 ± 0.02
**O(PA)$_8$**	1.7 ± 0.1	1.1 ± 0.2

Values are mean ± s.d. using n = 5 independent experiments. The other experimental conditions were the same as those stated in **Experimental Section**.

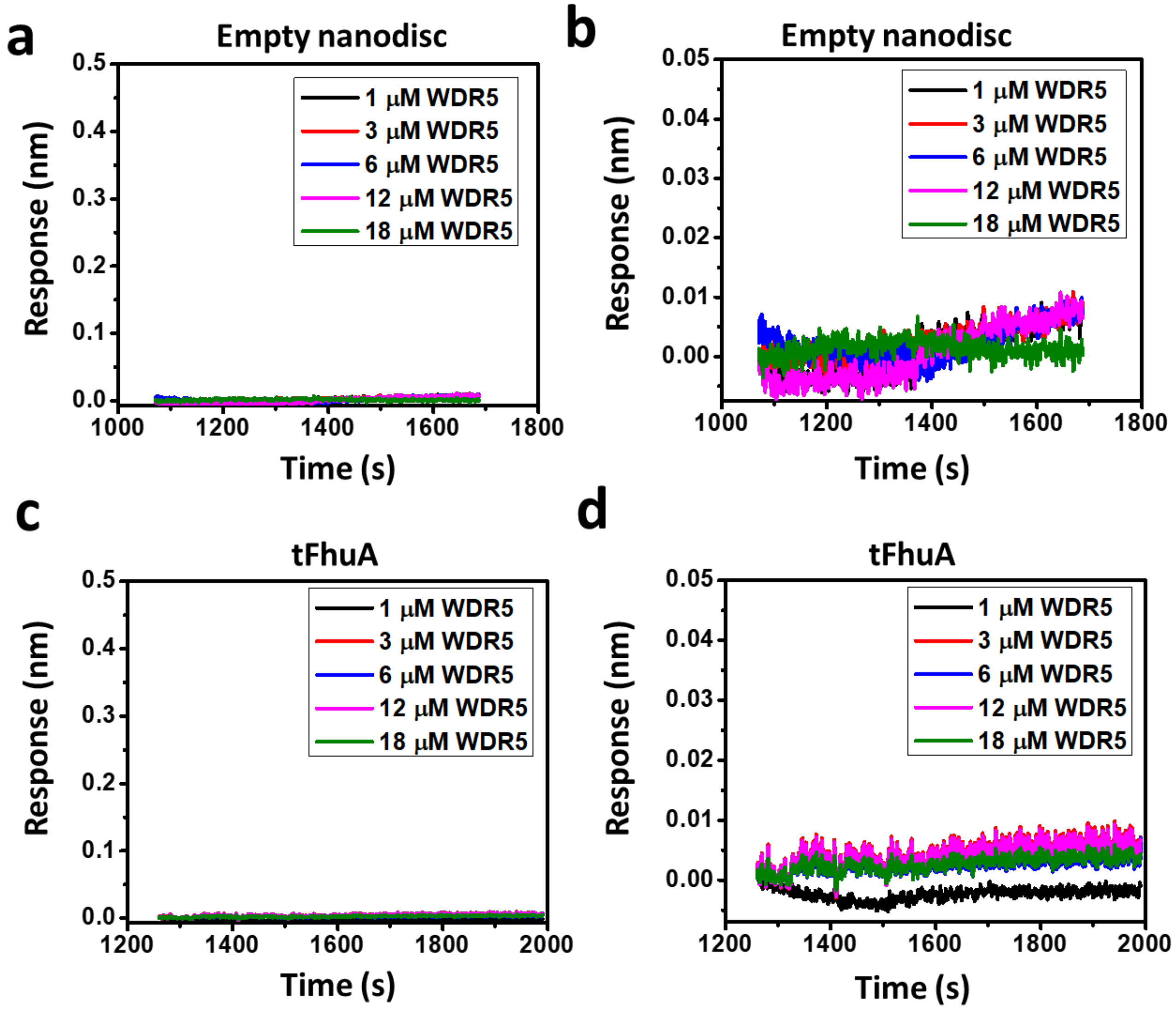

<u>Supplementary Figure S4.</u> ND-BLI sensorgrams of negative- and positive-control experiments. (a) 15 nM biotin-tagged empty ND made from lipid and membrane scaffold protein (MSP) was loaded onto streptavidin (SA) sensors for 5 min. Individual binding curves are indicated for [WDR5] from 1 μM to 18 μM. **(b)** The same ND-BLI measurements as described in (a) but zoomed in on the y-axis for an easier observation. **(c)** The same measurements as in (a) but conducted with an ND-reconstituted tFhuA nanopore. (**d**) The same measurements as in (c) but zoomed in on the y-axis for an easier observation.

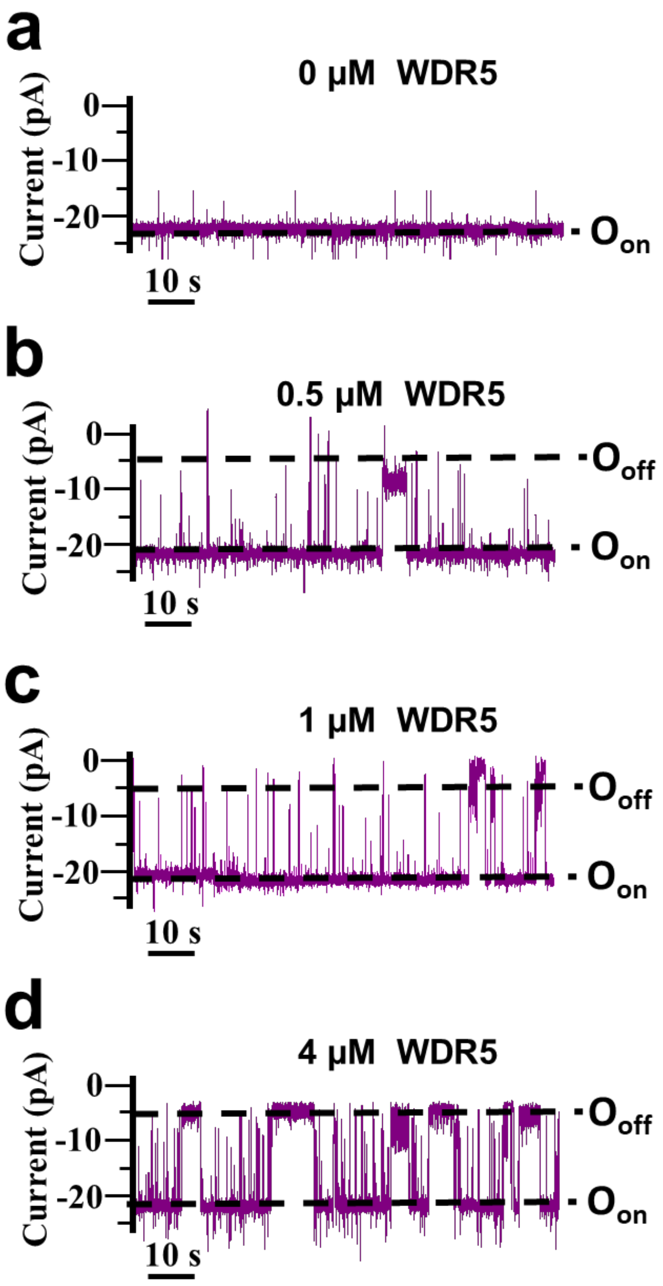

<u>Supplementary Figure S5.</u> Representative single-channel electrical traces of O(GGS)$_4$ in the presence of various concentrations of WDR5. The O_{on} and O_{off} levels correspond to the WDR5-released and WDR5-captured substates, respectively. **(a)** A representative single-channel electrical trace acquired with a O(GGS)$_4$ nanopore sensor without WDR5. (**b**)The same as (a) but in the presences of 0.5 μM. (**c**) The same as (a) but in the presences of 1 μM WDR5. (**d**) The same as (a) but in the presences of 4 μM WDR5. All electrical recordings were conducted at a transmembrane potential of –20 mV. The signal was low-pass filtered at 100 Hz using an 8-pole Bessel filter**.**

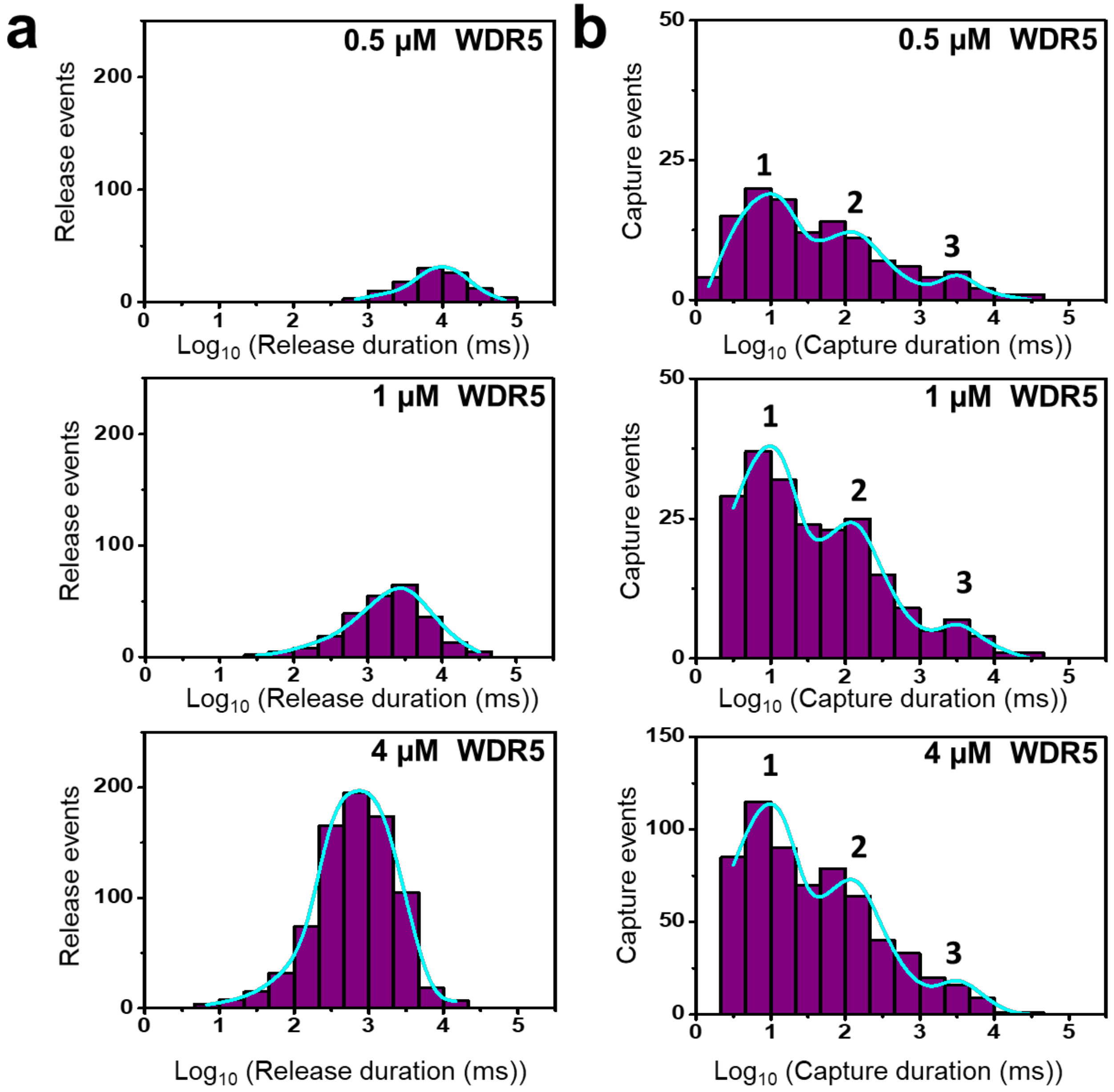

Supplementary Figure S6. Duration histograms of the WDR5-released and WDR5-captured events recorded with the O(GGS)$_4$ nanopore. (a) Histograms of WDR5-released event durations, whose values (mean ± s.e.m.) were 7.9 ± 1.8 s (number of events: N = 155), 3.8 ± 0.4 s (N = 204), and 1.0 ± 0.2 s (N = 856) at 0.5, 1, and 4 μM WDR5, respectively. **(b)** Histograms of WDR5-captured event durations. The cumulative fits are marked in cyan. All three binding events are numerically labeled. Here, 1, 2, and 3 indicate the short-, medium-, and long-lived binding events, respectively. For 0.5 μM WDR5, they (mean ± s.e.m.) were 0.009 ± 0.002 s, 0.14 ± 0.01 s, and 1.9 ± 0.6 s, respectively (N = 168). For 1 μM WDR5, they (mean ± s.e.m.) were 0.012 ± 0.002 s, 0.12 ± 0.03 s, and 1.1 ± 0.1 s, respectively (N = 211). For 4 μM WDR5, they (mean ± s.e.m.) were 0.011 ± 0.002 s, 0.13 ± 0.03 s, and 1.5 ± 0.3 s, respectively (N = 861). This data is from the representative trace of a reconstituted nanopore (**Supplementary Figure S5**).

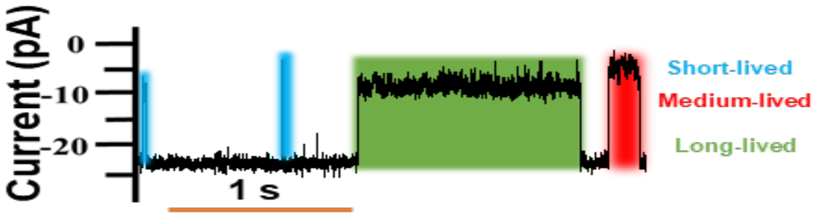

Supplementary Figure S7. The three different subpopulations of WDR5-MLL4$_{Win}$ interactions. An electrical recording with extended time frame to show the distribution of the three binding events. The short-lived events (1) are labeled in blue. The medium-lived event (2) is labeled in red and the long-lived event (3) in green. This recording was conducted at a transmembrane potential of –20 mV. The electrical signal was low-pass filtered at 100 Hz using an 8-pole Bessel filter.

Supplementary Table S4. The probability distribution of the three binding events of WDR5-MLL4$_{Win}$ interactions recorded with the O(GGS)$_4$ sensor. These events were differentiated by their WDR5-captured durations. Individual experimental values were derived using event-list histograms in ClampFit (Axon). Fittings of event histograms were conducted in a semilogarithmic representation.[15] The maximum likelihood method[16, 17] and logarithm likelihood ratio (LLR) tests[18-20] were utilized to determine the best multi-exponential probability distribution model (**Experimental Section**).

Nanopore Sensor	[WDR5]	P_1	P_2	P_3
**O(GGS)$_4$**	0.5	0.55 ± 0.04	0.31 ± 0.08	0.14 ± 0.06
	1	0.52 ± 0.07	0.25 ± 0.06	0.13 ± 0.05
	2	0.58 ± 0.06	0.28 ± 0.10	0.14 ± 0.02
	4	0.58 ± 0.05	0.29 ± 0.11	0.10 ± 0.04

Values represent mean ± s.d. obtained from n = 4 independently reconstituted nanopore sensors. The other experimental conditions were the same as those stated in **Experimental Section**.

<u>Supplementary Table S5.</u> WDR5-released and WDR5-captured event durations obtained with the O(GGS)$_4$ nanopore. Subscript "i" is 1, 2, and 3 are assigned to the short-, medium-, and long-lived binding events, respectively. τ_{on} are mean values of the WDR5-released durations. $\tau_{on-1} = \tau_{on} / P_1$, where P_1 is the probability of short-lived binding events. τ_{on-2} and τ_{on-3} were calculated similarly. The mean values of those probabilities are listed in **Supplementary Table S4**. τ_{off-i} values are the mean values of the WDR5-captured durations. All histogram fittings were conducted using a semilogarithmic representation.[15] The maximum likelihood method[16, 17] and logarithm likelihood ratio (LLR) tests[18-20] were utilized to determine the best multi-exponential probability distribution model (**Experimental Section**).

Nanopore Sensor	[WDR5]	τ_{on} (s)	τ_{on-1} (s)	τ_{on-2} (s)	τ_{on-3} (s)	$\tau_{off-1} \times 10^3$ (s)	τ_{off-2} (s)	τ_{off-3} (s)
O(GGS)$_4$	0.5	7.8 ± 2.1	13 ± 3	26 ± 4	50 ± 3	8 ± 2	0.10 ± 0.03	1.8 ± 0.1
	1	3.4 ± 0.4	6.3 ± 0.6	11 ± 3	24 ± 2	13 ± 2	0.12 ± 0.02	2.1 ± 0.4
	2	1.8 ± 0.8	3.1 ± 0.5	6.4 ± 1.5	13 ± 3	10 ± 4	0.14 ± 0.03	1.7 ± 0.5
	4	0.7 ± 0.2	1.3 ± 0.3	3.0 ± 1.1	6.2 ± 2.0	14 ± 3	0.14 ± 0.05	1.3 ± 0.6

Values represent mean ± s.d. obtained from $n = 4$ independently reconstituted nanopore sensors. The other experimental conditions were the same as those stated in **Experimental Section**.

<u>Supplementary Table S6.</u> The association and dissociation rate constants of the three WDR5-MLL4$_{Win}$ binding events noted with O(GGS)$_4$. The association rate constants of the short-lived events, k_{on-1}, were determined using the equation: $k_{on-1} = 1/(\tau_{on-1}[WDR5])$. The association rate constants of the medium-lived events, k_{on-2}, and long-lived events, k_{on-3}, were determined in the same way. τ_{on-i} values are provided in **Supplementary Table S5**. The dissociation rate constants of the short-lived events were determined using the equation: $k_{off-1} = 1/\tau_{off-1}$. We used the same approach for determining the dissociation rate constants of the medium-lived events, k_{off-2}, and long-lived events, k_{off-3}. τ_{off-i} values are listed in **Supplementary Table S5.**

Nanopore Sensor	[WDR5]	$k_{on-1} \times 10^{-5}$ ($M^{-1}s^{-1}$)	$k_{on-2} \times 10^{-4}$ ($M^{-1}s^{-1}$)	$k_{on-3} \times 10^{-4}$ ($M^{-1}s^{-1}$)	k_{off-1} (s^{-1})	k_{off-2} (s^{-1})	k_{off-3} (s^{-1})
O(GGS)$_4$	0.5	1.6 ± 0.10	8.0 ± 1.7	3.2 ± 1.1	84 ± 5.6	8.9 ± 0.60	0.84 ± 0.02
	1	1.4 ± 0.16	7.1 ± 1.4	4.5 ± 0.8	91 ± 6.4	9.4 ± 0.44	0.77 ± 0.04
	2	1.6 ± 0.20	7.8 ± 1.8	3.9 ± 1.0	87 ± 8.2	9.9 ± 0.30	0.77 ± 0.03
	4	2.1 ± 0.15	8.3 ± 2.1	3.4 ± 0.9	81 ± 5.1	9.5 ± 0.37	0.79 ± 0.06

Values represent mean ± s.d. obtained from $n = 4$ independently reconstituted nanopore sensors. The other experimental conditions were the same as those stated in **Experimental Section**.

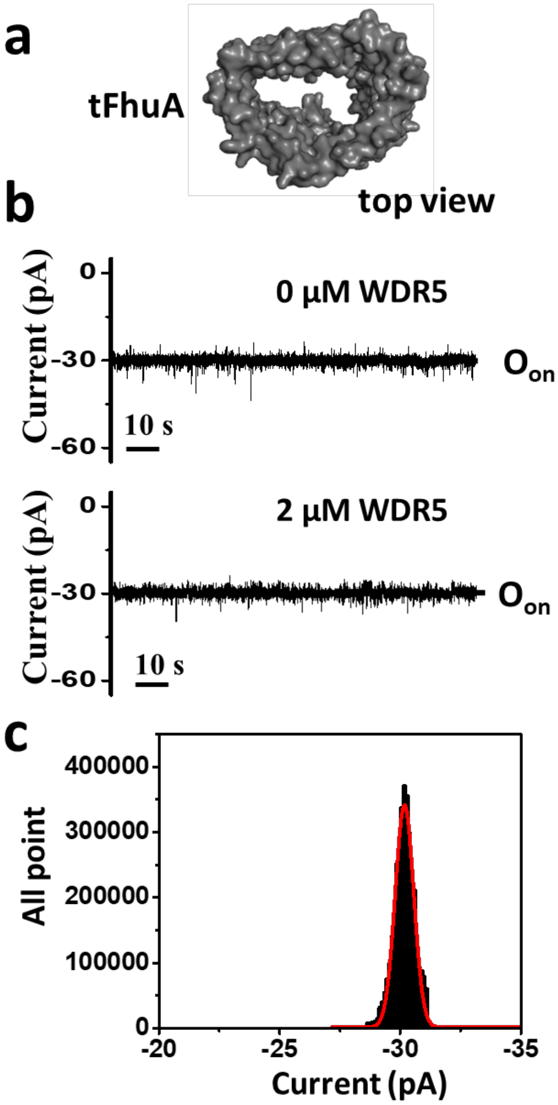

<u>Supplementary Figure S8.</u> Characterizing the unmodified tFhuA nanopore sensor. (a) A top view cartoon of the unmodified tFhuA. (**b**) a representative single-channel electrical trace recorded with an unmodified tFhuA nanopore[5, 6] in the absence of WDR5.[11, 12] The O_{on} level corresponds to the WDR5-released substate. The same single-channel electrical trace as above but in the presence of 2 µM target WDR5 added to the *cis* compartment. No current blockades were noted. (**c**)An all point histogram showing the current distribution for the unmodified tFhuA open current. All electrical recordings were conducted at a transmembrane potential of –20 mV. The electrical signal was low-pass filtered at a frequency of 100 Hz using an 8-pole Bessel filter.

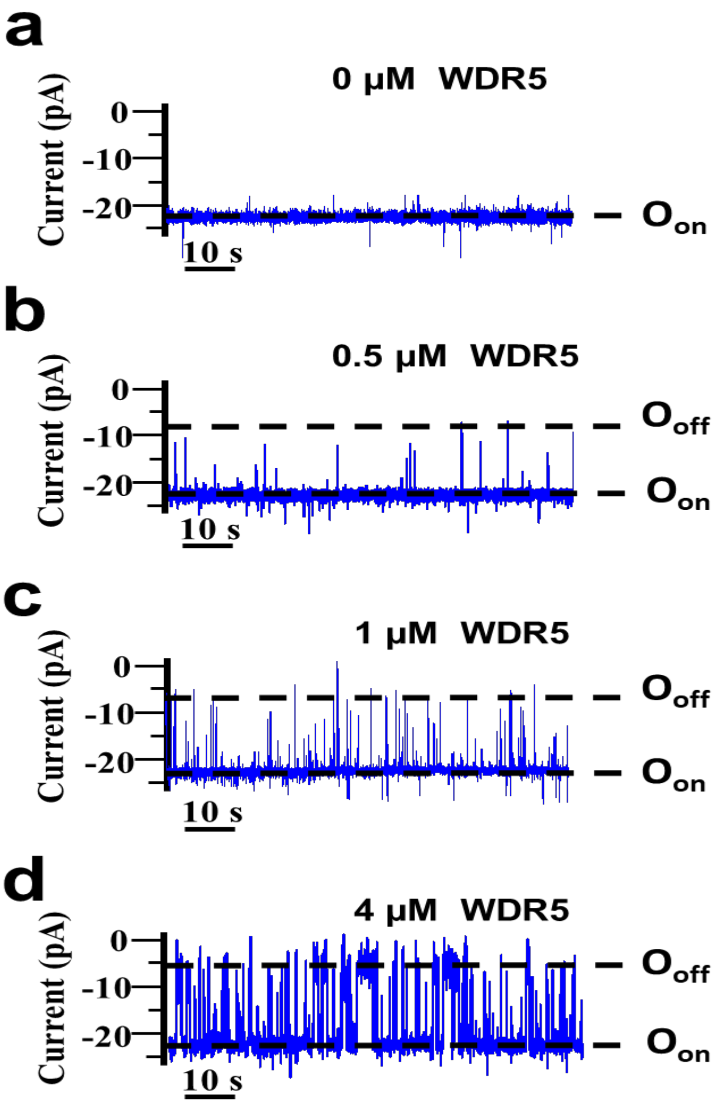

<u>Supplementary Figure S9.</u> Representative single-channel electrical traces of O(PA)$_6$ at various WDR5 concentrations. The O$_{on}$ and O$_{off}$ levels correspond to the WDR5-released and WDR5-captured substates, respectively. **(a)** A representative single-channel electrical trace acquired with a O(PA)$_6$ sensor in the absence of WDR5. (**b**)The same as in (a) but in the presence of 0.5 µM. (**c**) The same as in (a) but in the presence of 1 µM WDR5. (**d**) The same as in (a) but in the presence of 4 µM WDR5. All electrical recordings were conducted at a transmembrane potential of –20 mV. Traces were low-pass filtered at 100 Hz using an 8-pole Bessel filter**.**

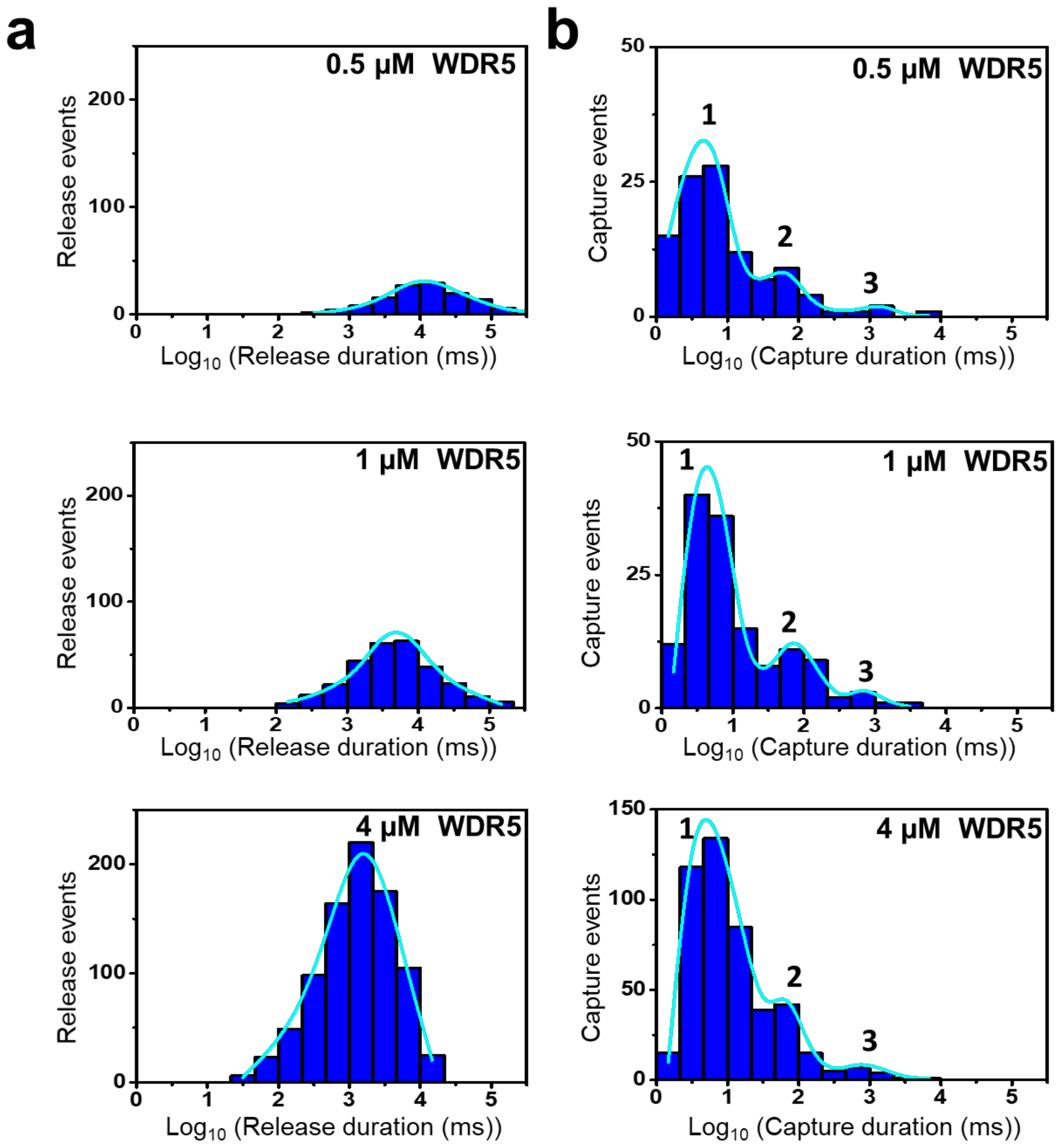

<u>Supplementary Figure S10.</u> Duration histograms of the WDR5-released and WDR5-captured events recorded with the O(PA)$_6$ nanopore. (a) Histograms of WDR5-released event durations, whose values (mean ± s.e.m.) were 12 ± 3 s (number of events: N = 99), 5.6 ± 0.6 s (N = 131), and 1.5 ± 0.6 s (N = 503) at 0.5, 1, and 4 μM WDR5, respectively. **(b)** Histograms of WDR5-captured event durations. The cumulative fits are marked in cyan. The three binding events are numerically labeled. Here, 1, 2, and 3 indicate the short-, medium-, and long-lived binding events, respectively. For 0.5 μM WDR5, they (mean ± s.e.m.) were 0.005 ± 0.001 s, 0.050 ± 0.008 s, and 1.1 ± 0.5 s, respectively (N = 102). For 1 μM WDR5, they (mean ± s.e.m.) were 0.007 ± 0.003 s, 0.04 ± 0.01 s, and 1.1 ± 0.4 s, respectively (N = 139). For 4 μM WDR5, they (mean ± s.e.m.) were 0.004 ± 0.001 s, 0.04 ± 0.01 s, and 1.0 ± 0.2 s, respectively (N = 516). This data is from the representative trace of a reconstituted nanopore (**Supplementary Figure S9**) .

Supplementary Table S7. The probability distribution of the three binding events of WDR5-MLL4$_{Win}$ interactions recorded with O(PA)$_6$ nanopores. These events were differentiated by their WDR5-captured durations. Individual experimental values were derived using event-list histograms in ClampFit (Axon). Fittings of event histograms were conducted in a semilogarithmic representation.[15] The maximum likelihood method[16, 17] and logarithm likelihood ratio (LLR) tests[18-20] were utilized to determine the best multi-exponential probability distribution model (**Experimental Section**).

Nanopore Sensor	[WDR5]	P_1	P_2	P_3
**O(PA)$_6$**	0.5	0.76 ± 0.05	0.26 ± 0.04	0.04 ± 0.01
	1	0.78 ± 0.08	0.20 ± 0.06	0.02 ± 0.01
	2	0.72 ± 0.08	0.26 ± 0.05	0.02 ± 0.01
	4	0.76 ± 0.10	0.22 ± 0.04	0.03 ± 0.01

Values represent mean ± s.d. obtained from n = 3 independently reconstituted nanopore sensors. The other experimental conditions were the same as those stated in **Experimental Section**.

Supplementary Table S8. WDR5-released and WDR5-captured event durations obtained with O(PA)$_6$ nanopores. Here, 1, 2, and 3 are assigned to the short-, medium-, and long-lived binding events, respectively. τ_{on} are mean values of the WDR5-released durations. $\tau_{on-1} = \tau_{on}/P_1$, where P_1 is the probability of short-lived binding events. τ_{on-2} and τ_{on-3} were calculated similarly. The mean values of those probabilities are listed in **Supplementary Table S7**. τ_{off-i} are the mean values of the WDR5-captured durations. All histogram fittings were conducted using a semilogarithmic representation.[15] The maximum likelihood method[16, 17] and logarithm likelihood ratio (LLR) tests[18-20] were utilized to determine the best multi-exponential probability distribution model (**Experimental Section**).

Nanopore Sensor	[WDR5]	τ_{on} (s)	τ_{on-1} (s)	τ_{on-2} (s)	τ_{on-3} (s)	$\tau_{off-1} \times 10^3$ (s)	τ_{off-2} (s)	τ_{off-3} (s)
**O(PA)$_6$**	0.5	11 ± 0.5	18 ± 0.4	49 ± 4	271 ± 9	4 ± 1	0.03 ± 0.01	1.1 ± 0.2
	1	6.1 ± 0.4	8.1 ± 0.3	23 ± 0.4	133 + 9	5 ± 1	0.05 ± 0.02	1.6 ± 0.4
	2	2.6 ± 0.3	3.8 ± 0.4	9.9 ± 0.6	65 ± 7	6 ± 2	0.05 ± 0.01	1.1 ± 0.3
	4	1.1 ± 0.5	1.7 ± 0.6	5.1 ± 0.3	31 ± 3	4 ± 2	0.04 ± 0.01	1.5 ± 0.4

Values represent mean ± s.d. obtained from n = 3 independently reconstituted nanopores for O(PA)$_6$. The other experimental conditions were the same as those stated in **Experimental Section**.

Supplementary Table S9. The association and dissociation rate constants of the three binding events observed with $O(PA)_6$. The association rate constants of the short-lived events, k_{on-1}, were determined using the equation: $k_{on-1} = 1/(\tau_{on-1}[WDR5])$. The association rate constants of the medium-lived events, k_{on-2}, and long-lived events, k_{on-3}, were determined in the same way. τ_{on-i} values are provided in **Supplementary Table S8**. The dissociation rate constants of the short-lived events were determined using the equation: $k_{off-1} = 1/\tau_{off-1}$. We used the same approach for determining the dissociation rate constants of the medium-lived events, k_{off-2}, and long-lived events, k_{off-3}. τ_{off-i} values are listed in **Supplementary Table S8**.

Nanopore Sensor	[WDR5]	$k_{on-1} \times 10^{-5}$ ($M^{-1}s^{-1}$)	$k_{on-2} \times 10^{-4}$ ($M^{-1}s^{-1}$)	$k_{on-3} \times 10^{-4}$ ($M^{-1}s^{-1}$)	k_{off-1} (s^{-1})	k_{off-2} (s^{-1})	k_{off-3} (s^{-1})
**$O(PA)_6$**	0.5	1.0 ± 0.2	5.6 ± 0.9	0.70 ± 0.06	178 ± 7	18 ± 3	0.77 ± 0.06
	1	1.5 ± 0.4	5.2 ± 1.1	0.81 ± 0.07	159 ± 10	23 ± 4	0.78 ± 0.01
	2	1.3 ± 0.2	4.8 ± 1.2	0.75 ± 0.05	170 ± 9	20 ± 2	0.77 ± 0.05
	4	1.7 ± 0.1	4.4 ± 1.1	0.74 ± 0.05	167 ± 6	21 ± 2	0.73 ± 0.02

Values are mean ± s.d. from $n = 3$ independently reconstituted nanopores. The other experimental conditions were the same as those stated in **Experimental Section**.

Supplementary Table S10. The association and dissociation rate constants of the three WDR5-$MLL4_{Win}$ binding events noted with $O(GGS)_4$ and $O(PA)_6$. k_{on} values were determined as the slopes of the linear fits in **Figure 3c**. k_{off} values are the axis intercepts of the horizontal line fits in **Figure 3d**.

Nanopore Sensor	$k_{on-1} \times 10^{-5}$ ($M^{-1}s^{-1}$)	$k_{on-2} \times 10^{-4}$ ($M^{-1}s^{-1}$)	$k_{on-3} \times 10^{-4}$ ($M^{-1}s^{-1}$)	k_{off-1} (s^{-1})	k_{off-2} (s^{-1})	k_{off-3} (s^{-1})
**$O(GGS)_4$**	1.4 ± 0.2	7.9 ± 0.4	4.0 ± 0.5	86 ± 3	9.2 ± 0.5	0.78 ± 0.02
**$O(PA)_6$**	1.6 ± 0.2	5.1 ± 0.2	0.80 ± 0.04	170 ± 8	18 ± 1	0.80 ± 0.02

Values represent mean ± s.d. obtained from $n = 4$ and $n = 3$ independently reconstituted nanopore sensors for $O(GGS)_4$ and $O(PA)_6$, respectively. The other experimental conditions were the same as those stated in **Experimental Section**.

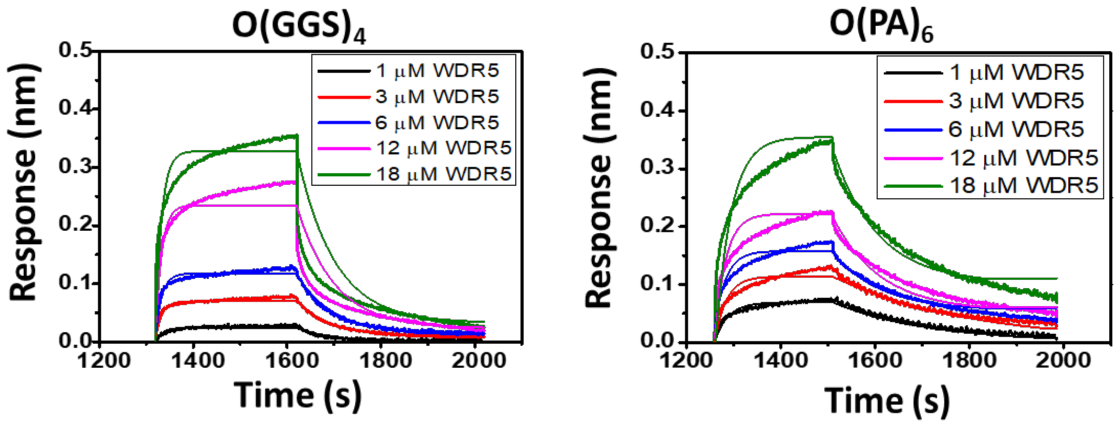

Supplementary Figure S11. Fits of the ND-BLI sensorgrams for $O(GGS)_4$ and $O(PA)_6$. The FortéBio Octet Data Analysis software (FortéBio) was used for the fitting of binding curves. The binding curves are in **Fig. 3e and Fig. 3f** . The curves of the association process were fitted using the following equation: $Y = Y_\infty - (Y_\infty - Y_0)exp(-k_{obs}t)$.[14] Here, Y_0 and Y_∞ are the response signals during the association process at zero and infinity times, respectively. t is the cumulative time of the association reaction. k_{obs} denotes the apparent first-order reaction rate constant of the association process. The dissociation process was fitted using the following equation:
$Y = Y_\infty + (Y_0 - Y_\infty)exp(-k_{off}t)$, where Y_0 and Y_∞ are the response signals during the dissociation process at zero and infinity times, respectively. k_{off} shows the dissociation rate constant. The fits are shown as thin lines with the corresponding color to the curve. Due to the elevated WDR5 concentration, [WDR5], at 12 μM and 18 μM we observe more response (signal) drift, which is the basis for some distinctions between the fits and binding curves.

Supplementary Table S11. ND-BLI-determined kinetic rate constants for $O(GGS)_4$ and $O(PA)_6$. The association and dissociation rate constants were inferred, as previously reported.[1]

Nanopore Sensor	k_{on} ($M^{-1}s^{-1}$) × 10^{-4}	k_{off} (s^{-1}) × 10^{2}
**$O(GGS)_4$**	2.0 ± 0.4	0.39 ± 0.04
**$O(PA)_6$**	1.1 ± 0.2	1.2 ± 0.1

Values are mean ± s.d. using n = 5 independent experiments for both constructs. The other experimental conditions were the same as those stated in **Experimental Section**.

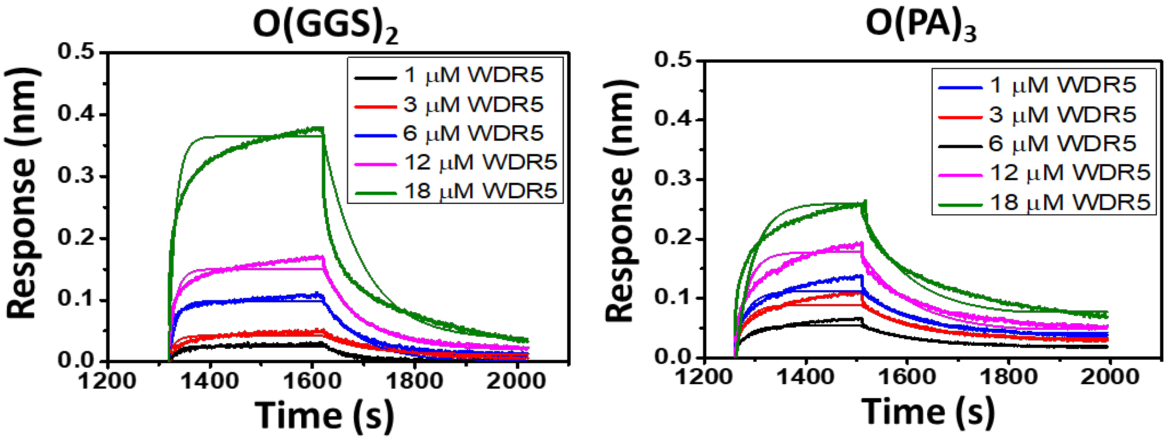

Supplementary Figure S12. Fits of the ND-BLI sensorgrams for O(GGS)$_2$ and **O(PA)$_3$.** The FortéBio Octet Data Analysis software (FortéBio) was used for the fitting of binding curves. The binding curves are in **Fig. 4a and Fig. 4b**. The curves of the association process were fitted using the following equation: $Y = Y_\infty - (Y_\infty - Y_0)exp(-k_{obs}t)$.[14] Here, Y_0 and Y_∞ are the response signals during the association process at zero and infinity times, respectively. t is the cumulative time of the association reaction. k_{obs} denotes the apparent first-order reaction rate constant of the association process. The dissociation process was fitted using the following equation:
$Y = Y_\infty + (Y_0 - Y_\infty)exp(-k_{off}t)$, where Y_0 and Y_∞ are the response signals during the dissociation process at zero and infinity times, respectively. k_{off} shows the dissociation rate constant. The fits are shown as thin lines with the corresponding color to the curve. Due to the elevated WDR5 concentration, [WDR5], at 12 μM and 18 μM we observe more response (signal) drift, which is the basis for some distinctions between the fits and binding curves.

Supplementary Table S12. ND-BLI-determined kinetic rate constants for O(GGS)$_2$ and O(PA)$_3$. The association and dissociation rate constants were inferred, as previously reported.[1]

Nanopore Sensor	k_{on} ($M^{-1}s^{-1}$) × 10^{-4}	k_{off} (s^{-1}) × 10^{2}
**O(GGS)$_2$**	1.9 ± 0.2	0.46 ± 0.02
**O(PA)$_3$**	0.60 ± 0.08	1.8 ± 0.2

Values are mean ± s.d. using n = 5 independent experiments for both sensors. The other experimental conditions were the same as those stated in **Experimental Section**.

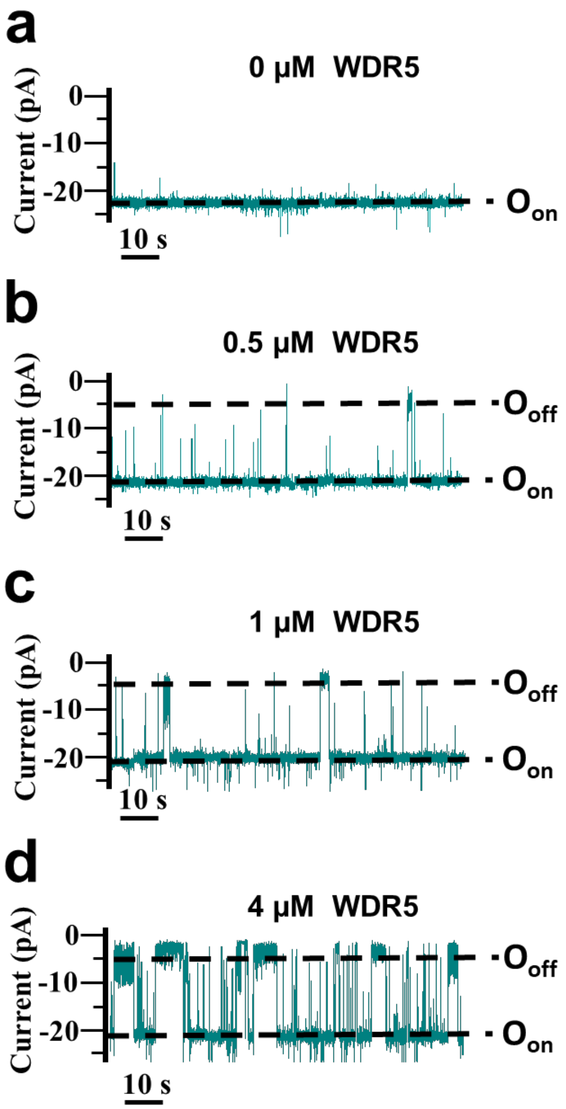

Supplementary Figure S13. Representative single-channel electrical traces of O(GGS)$_2$ recorded at various WDR5 concentrations. The O_{on} and O_{off} levels correspond to the WDR5-released and WDR5-captured substates, respectively. **(a)** A representative single-channel electrical trace acquired with an O(GGS)$_2$ nanopore sensor in the absence of WDR5. (**b**) The same as (a) but in the presences of 0.5 µM WDR5. (**c**) The same as (a) but in the presences of 1 µM WDR5. (**d**) The same as (a) but in the presence of 4 µM WDR5. All electrical recordings were conducted at a transmembrane potential of –20 mV. The electrical signal was low-pass filtered at 100 Hz using an 8-pole Bessel filter.

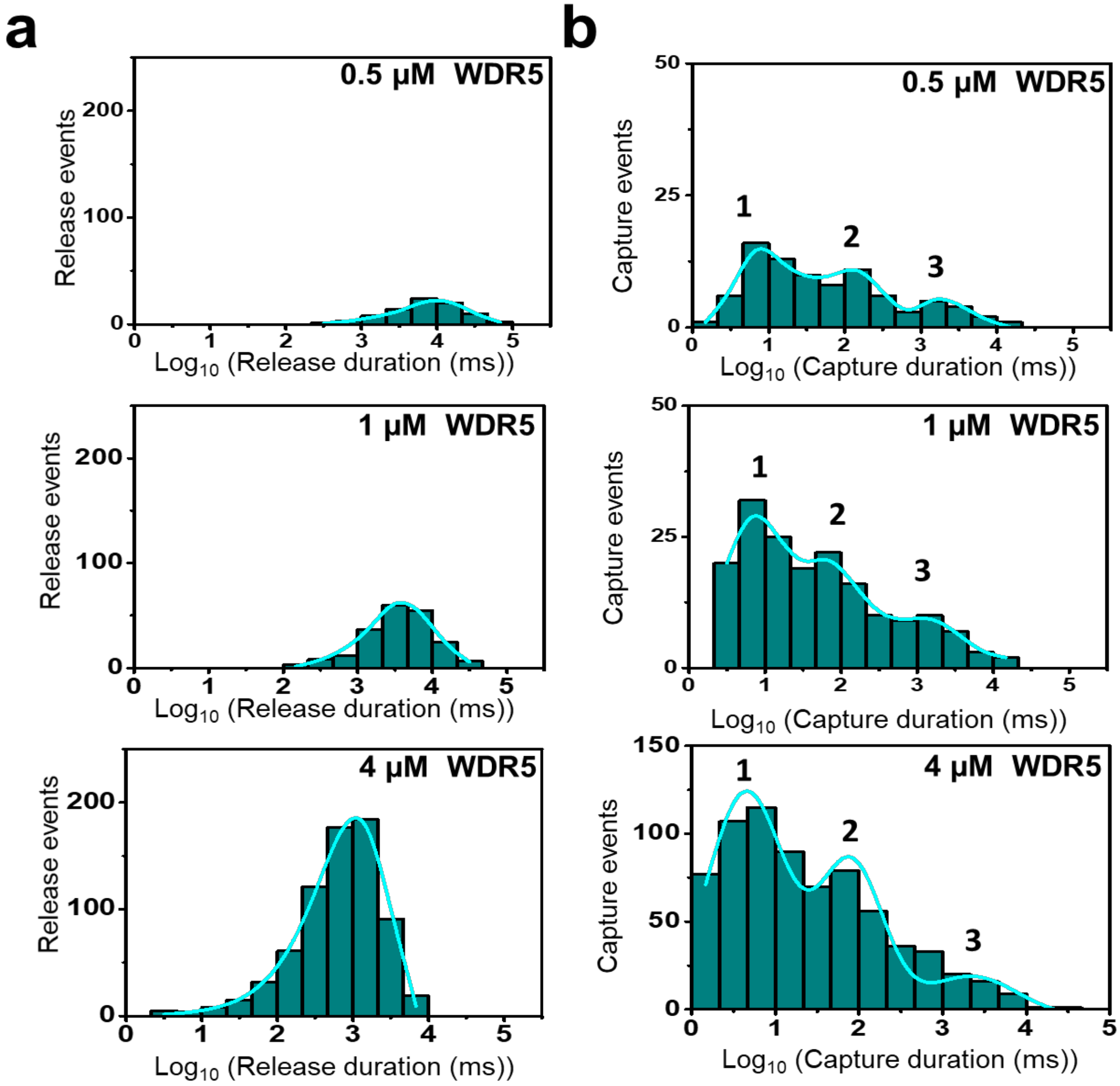

<u>Supplementary Figure S14.</u> **Duration histograms of the WDR5-released and WDR5-captured events recorded with the O(GGS)$_2$ nanopore. (a)** Histograms of WDR5-released event durations, whose values (mean ± s.e.m.) were 8.5 ± 1.3 s (number of events: $N = 102$), 4.2 ± 0.3 s ($N = 185$), and 1.3 ± 0.5 s ($N = 746$) at 0.5, 1, and 4 µM WDR5, respectively. Single-exponential fits in semilogarithmic representations are indicated in cyan. **(b)** Histograms of WDR5-captured event durations for 0.5, 1, and 4 µM WDR5, respectively. The cumulative fits are marked in cyan. All three binding events are numerically labeled. Here, 1, 2, and 3 indicate the short-, medium- and long-lived binding events, respectively. For 0.5 µM WDR5, they (mean ± s.e.m.) were 0.010 ± 0.002 s, 0.100 ± 0.008 s, and 1.2 ± 0.4 s, respectively ($N = 109$). For 1 µM WDR5, they (mean ± s.e.m.) were 0.008 ± 0.003 s, 0.13 ± 0.01 s, and 1.9 ± 0.1 s, respectively ($N = 177$). For 4 µM WDR5, they (mean ± s.e.m.) were 0.012 ± 0.002 s, 0.18 ± 0.01 s, and 1.8 ± 0.2 s, respectively ($N = 754$). This data is from the representative trace of a reconstituted nanopore (**Supplementary Figure S13**).

<u>Supplementary Table S13.</u> The probability distribution of the three binding events of WDR5-MLL4$_{Win}$ interactions recorded with the O(GGS)$_2$ sensor. These events were differentiated by their WDR5-captured durations. Individual experimental values were derived using event-list histograms in ClampFit (Axon). Fittings of event histograms were conducted in a semilogarithmic representation.[15] The maximum likelihood method[16, 17] and logarithm likelihood ratio (LLR) tests[18-20] were utilized to determine the best multi-exponential probability distribution model (**Experimental Section**).

Nanopore Sensor	[WDR5]	P_1	P_2	P_3
**O(GGS)$_2$**	0.5	0.58 ± 0.04	0.29 ± 0.06	0.13 ± 0.04
	1	0.56 ± 0.04	0.28 ± 0.05	0.15 ± 0.05
	2	0.57 ± 0.05	0.29 ± 0.09	0.14 ± 0.03
	4	0.59 ± 0.06	0.27 ± 0.06	0.13 ± 0.04

Values represent mean ± s.d. obtained from n = 6 independently reconstituted nanopore sensors. The other experimental conditions were the same as those stated in **Experimental Section**.

<u>Supplementary Table S14</u>. WDR5-released and WDR5-captured event durations obtained with the O(GGS)$_2$ nanopore sensor. 1, 2, and 3 are assigned to the short-, medium-, and long-lived binding events, respectively. τ_{on} are mean values of the WDR5-released durations. $\tau_{on\text{-}1} = \tau_{on}/P_1$, where P_1 is the probability of short-lived binding events. $\tau_{on\text{-}2}$ and $\tau_{on\text{-}3}$ were calculated similarly. The mean values of those probabilities are listed in **Supplementary Table S13**. $\tau_{off\text{-}i}$ values are the mean values of the WDR5-captured durations. All histogram fittings were conducted using a semilogarithmic representation.[15] The maximum likelihood method[16, 17] and logarithm likelihood ratio (LLR) tests[18-20] were utilized to determine the best multi-exponential probability distribution model (**Experimental Section**).

Nanopore Sensor	[WDR5]	τ_{on} (s)	$\tau_{on\text{-}1}$ (s)	$\tau_{on\text{-}2}$ (s)	$\tau_{on\text{-}3}$ (s)	$\tau_{off\text{-}1} \times 10^3$ (s)	$\tau_{off\text{-}2}$ (s)	$\tau_{off\text{-}3}$ (s)
**O(GGS)$_2$**	0.5	8.5 ± 1.2	14 ± 5	33 ± 9	55 ± 6	11 ± 3	0.11 ± 0.01	1.4 ± 0.35
	1	4.3 ± 0.2	7.2 ± 0.5	16 ± 2	28 ± 3	9 ± 2	0.12 ± 0.04	1.9 ± 0. 24
	2	2.0 ± 0.6	3.5 ± 0.8	7.1 ± 1.6	15 ± 4.1	10 ± 2	0.12 ± 0.05	1.6 ± 0.30
	4	1.3 ± 0.5	2.1 ± 0.7	4.3 ± 1.5	8.5 ± 2.6	8 ± 4	0.15 ± 0.03	2.2 ± 0.50

Values represent mean ± s.d. obtained from n = 6 independently reconstituted nanopore sensors. The other experimental conditions were the same as those stated in **Experimental Section**.

<u>**Supplementary Table S15.**</u> **The association and dissociation rate constants of the three WDR5-MLL4$_{Win}$ binding events noted with O(GGS)$_2$.** The association rate constants of the short-lived events, k_{on-1}, were determined using the equation: $k_{on-1} = 1/(\tau_{on-1}[WDR5])$. The association rate constants of the medium-lived events, k_{on-2}, and long-lived events, k_{on-3}, were determined in the same way. τ_{on-i} values are provided in **Supplementary Table S14**. The dissociation rate constants of the short-lived events were determined using the equation: $k_{off-1} = 1/\tau_{off-1}$. We used the same approach for determining the dissociation rate constants of the medium-lived events, k_{off-2}, and long-lived events, k_{off-3}. τ_{off-i} values are listed in **Supplementary Table S14.**

Nanopore Sensor	[WDR5]	$k_{on-1} \times 10^{-5}$ ($M^{-1}s^{-1}$)	$k_{on-2} \times 10^{-4}$ ($M^{-1}s^{-1}$)	$k_{on-3} \times 10^{-4}$ ($M^{-1}s^{-1}$)	k_{off-1} (s^{-1})	k_{off-2} (s^{-1})	k_{off-3} (s^{-1})
**O(GGS)$_2$**	0.5	1.5 ± 0.10	7.3 ± 1.6	3.6 ± 0.75	90 ± 4.9	9.6 ± 4.6	0.72 ± 0.05
	1	1.4 ± 0.09	7.0 ± 1.1	4.1 ± 0.57	88 ± 7.5	8.9 ± 2.3	0.91 ± 0.11
	2	1.4 ± 0.10	7.1 ± 1.8	3.6 ± 0.80	86 ± 6.1	9.2 ± 2.5	0.78 ± 0.06
	4	1.6 ± 0.08	6.8 ± 1.3	3.2 ± 0.60	88 ± 5.8	11 ± 3.1	0.82 ± 0.09

Values represent mean ± s.d. obtained from $n = 6$ independently reconstituted nanopore sensors. The other experimental conditions were the same as those stated in **Experimental Section**.

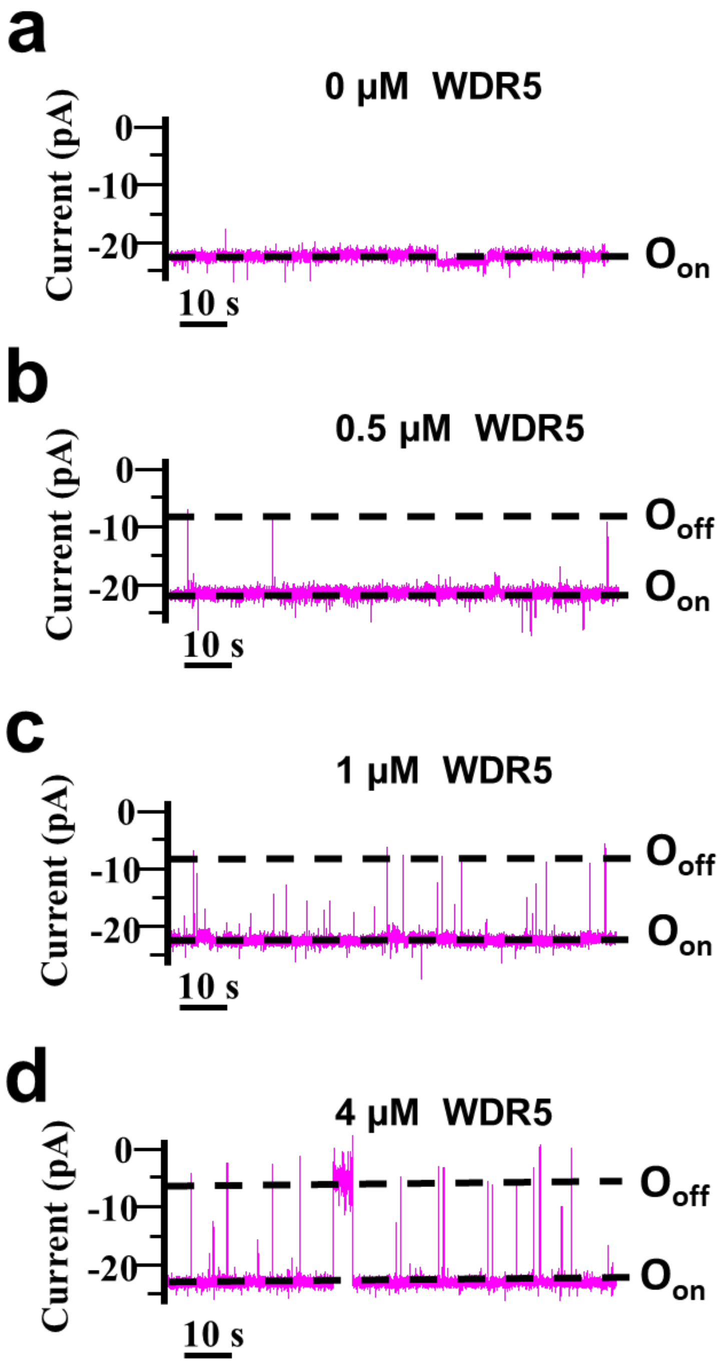

<u>Supplementary Figure S15.</u> Representative single-channel electrical traces of $O(PA)_3$ in the presence of various concentrations of WDR5. The O_{on} and O_{off} are WDR5-released and WDR5-captured substates, respectively. **(a)** A representative single-channel electrical trace acquired with a $O(PA)_3$ nanopore sensor in the absence of WDR5. (**b**) The same as (a) but in the presences of 0.5 µM WDR5. (**c**) The same as (a) but in the presences of 1 µM WDR5. (**d**) The same as (a) but in the presences of 4 µM WDR5. All electrical recordings were conducted at a transmembrane potential of –20 mV. Traces were low-pass filtered at 100 Hz using an 8-pole Bessel filter.

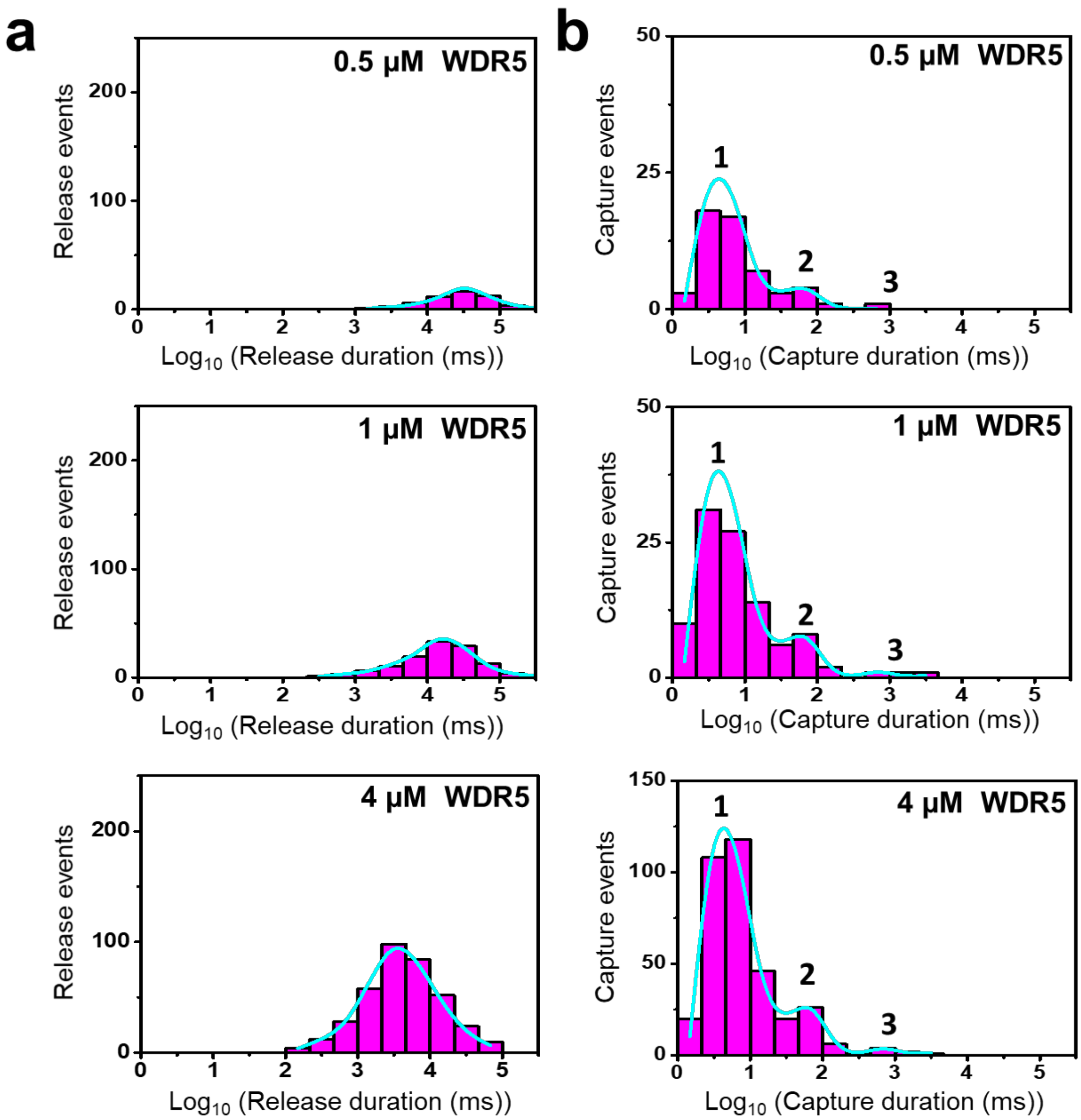

<u>Supplementary Figure S16.</u> Duration histograms of the WDR5-released and WDR5-captured events recorded with O(PA)$_3$. (a) Histograms of WDR5-released event durations, whose values (mean ± s.e.m.) were 45 ± 7 s (number of events: $N = 47$), 22 ± 4 s ($N = 99$), and 5.4 ± 0.9 s ($N = 407$) at 0.5, 1, and 4 μM WDR5, respectively. **(b)** Histograms of WDR5-captured event durations. The cumulative fits are marked in cyan. All three binding events are numerically labeled. 1, 2, and 3 indicate the short-, medium-, and long-lived binding events, respectively. For 0.5 μM WDR5, they (mean ± s.e.m.) were 0.005 ± 0.001 s, 0.04 ± 0.01 s, and 1.3 ± 0.4 s, respectively ($N = 42$). For 1 μM WDR5, they (mean ± s.e.m.) were 0.004 ± 0.001 s, 0.05 ± 0.01 s, and 1.0 ± 0.3 s, respectively ($N = 91$). For 4 μM WDR5, they (mean ± s.e.m.) were 0.005 ± 0.001 s, 0.06 ± 0.01 s, and 1.1 ± 0.2 s, respectively ($N = 415$). This data is from the representative trace of a reconstituted nanopore (**Supplementary Figure S15**).

Supplementary Table S16. The probability distribution of the three binding events of WDR5-MLL4$_{Win}$ interactions recorded with O(PA)$_3$ nanopores. These events were differentiated by their WDR5-captured durations. Individual experimental values were derived using event-list histograms in ClampFit (Axon). Fittings of event histograms were conducted in a semilogarithmic representation.[15] The maximum likelihood method[16, 17] and logarithm likelihood ratio (LLR) tests[18-20] were utilized to determine the best multi-exponential probability distribution model (**Experimental Section**).

Nanopore Sensor	[WDR5]	P_1	P_2	P_3
**O(PA)$_3$**	0.5	0.75 ± 0.08	0.23 ± 0.04	0.03 ± 0.01
	1	0.77 ± 0.07	0.20 ± 0.05	0.03 ± 0.01
	2	0.73 ± 0.10	0.25 ± 0.03	0.02 ± 0.01
	4	0.75 ± 0.10	0.21 ± 0.05	0.04 ± 0.01

Values represent mean ± s.d. obtained from n = 3 independently reconstituted nanopore sensors. The other experimental conditions were the same as those stated in **Experimental Section**.

Supplementary Table S17. WDR5-released and WDR5-captured event durations obtained with O(PA)$_3$ nanopores. Here, 1, 2, and 3 are assigned to the short-, medium-, and long-lived binding events, respectively. τ_{on} are mean values of the WDR5-released durations. $\tau_{on-1} = \tau_{on} / P_1$, where P_1 is the probability of short-lived binding events. τ_{on-2} and τ_{on-3} were calculated similarly. The mean values of those probabilities are listed in **Supplementary Table S16.** τ_{off-i} are the mean values of the WDR5-captured durations. All histogram fittings were conducted using a semilogarithmic representation.[15] The maximum likelihood method[16, 17] and logarithm likelihood ratio (LLR) tests[18-20] were utilized to determine the best multi-exponential probability distribution model (**Experimental Section**).

Nanopore Sensor	[WDR5]	τ_{on} (s)	τ_{on-1} (s)	τ_{on-2} (s)	τ_{on-3} (s)	$\tau_{off-1} \times 10^3$ (s)	τ_{off-2} (s)	τ_{off-3} (s)
**O(PA)$_3$**	0.5	53 ± 5	71 ± 4	171 ± 6	NA	5 ± 1	0.03 ± 0.01	1.6 ± 0.3
	1	24 ± 3	35 ± 6	80 ± 9	NA	6 ± 2	0.05 ± 0.02	1.1 ± 0.5
	2	11 + 5	15 ± 3	42 ± 8	490 ± 5	4 ± 1	0.03 ± 0.01	1.0 ± 0.2
	4	7 ± 4	10 ± 2	26 ± 5	267 ± 6	4 ± 2	0.04 ± 0.01	1.7 ± 0.4

Values represent mean ± s.d. obtained from n = 3 independently reconstituted nanopores for O(PA)$_3$. NA stands for the lack of statistically significant data. The other experimental conditions were the same as those stated in **Experimental Section**.

<u>Supplementary Table S18.</u> The association and dissociation rate constants of the three binding events observed with $O(PA)_3$. The association rate constants of the short-lived events, k_{on-1}, were determined using the equation: $k_{on-1} = 1/(\tau_{on-1}[WDR5])$. The association rate constants of the medium-lived events, k_{on-2}, and long-lived events, k_{on-3}, were determined in the same way. τ_{on-i} values are provided in **Supplementary Table S17**. The dissociation rate constants of the short-lived events were determined using the equation: $k_{off-1} = 1/\tau_{off-1}$. We used the same approach for determining the dissociation rate constants of the medium-lived events, k_{off-2}, and long-lived events, k_{off-3}. τ_{off-i} values are listed in **Supplementary Table S17**.

Nanopore Sensor	[WDR5]	$k_{on-1} \times 10^{-5}$ ($M^{-1}s^{-1}$)	$k_{on-2} \times 10^{-4}$ ($M^{-1}s^{-1}$)	$k_{on-3} \times 10^{-4}$ ($M^{-1}s^{-1}$)	k_{off-1} (s^{-1})	k_{off-2} (s^{-1})	k_{off-3} (s^{-1})
**$O(PA)_3$**	0.5	0.35 ± 0.12	1.3 ± 0.2	0.09 ± 0.04	234 ± 13	30 ± 6	0.74 ± 0.02
	1	0.41 ± 0.08	1.5 ± 0.3	0.18 ± 0.05	207 ± 11	33 ± 5	0.79 ± 0.03
	2	0.36 ± 0.10	1.3 ± 0.3	0.11 ± 0.02	210 ± 10	34 ± 1	0.79 ± 0.04
	4	0.39 ± 0.07	1.6 ± 0.5	0.11 ± 0.03	207 ± 10	31 ± 4	0.71 ± 0.05

Values are mean ± s.d. from $n = 3$ independently reconstituted nanopores. The other experimental conditions were the same as those stated in **Experimental Section**.

<u>Supplementary Table S19.</u> The association and dissociation rate constants of the three WDR5-$MLL4_{Win}$ binding events noted with $O(GGS)_2$ and $O(PA)_3$. k_{on} values were determined as the slopes of the linear fits in **Figure 4c**. k_{off} values are the axis intercepts of the horizontal line fits in **Figure 4d**.

Nanopore Sensor	$k_{on-1} \times 10^{-5}$ ($M^{-1}s^{-1}$)	$k_{on-2} \times 10^{-4}$ ($M^{-1}s^{-1}$)	$k_{on-3} \times 10^{-4}$ ($M^{-1}s^{-1}$)	k_{off-1} (s^{-1})	k_{off-2} (s^{-1})	k_{off-3} (s^{-1})
**$O(GGS)_2$**	1.1 ± 0.2	6.9 ± 0.4	3.5 ± 0.7	88 ± 4	9.4 ± 0.4	0.77 ± 0.03
**$O(PA)_3$**	0.29 ± 0.09	1.0 ± 0.2	0.20 ± 0.04	220 ± 4	35 ± 1	0.79 ± 0.01

Values represent mean ± s.d. obtained from $n = 6$ and n = 5 independently reconstituted nanopore sensors for $O(GGS)_2$ and $O(PA)_3$, respectively. The other experimental conditions were the same as those stated in **Experimental Section**.

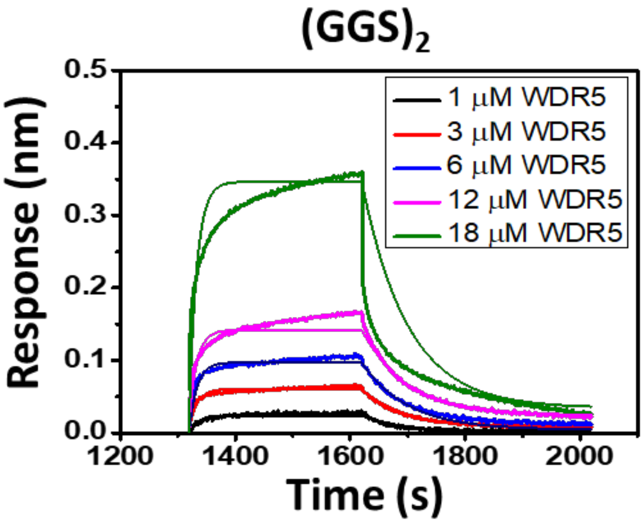

Supplementary Figure S17. Fits of the ND-BLI sensorgrams for $(GGS)_2$. The FortéBio Octet Data Analysis software (FortéBio) was used for the fitting of binding curves. The binding curves are in **Fig. 5c**. The curves of the association process were fitted using the following equation: $Y = Y_\infty - (Y_\infty - Y_0)exp(-k_{obs}t)$.[14] Here, Y_0 and Y_∞ are the response signals during the association process at zero and infinity times, respectively. t is the cumulative time of the association reaction. k_{obs} denotes the apparent first-order reaction rate constant of the association process. The dissociation process was fitted using the following equation:

$Y = Y_\infty + (Y_0 - Y_\infty)exp(-k_{off}t)$, where Y_0 and Y_∞ are the response signals during the dissociation process at zero and infinity times, respectively. k_{off} shows the dissociation rate constant. The fits are shown as thin lines with the corresponding color to the curve. Due to the elevated WDR5 concentration, [WDR5], at 12 μM and 18 μM we observe more response (signal) drift, which is the basis for some distinctions between the fits and binding curves.

Supplementary Table S20. ND-BLI-determined kinetic rate constants for $(GGS)_2$. The association and dissociation rate constants were inferred, as previously reported.[1]

Nanopore Sensor	k_{on} ($M^{-1}s^{-1}$) × 10^{-4}	k_{off} (s^{-1}) × 10^{2}
**$(GGS)_2$**	1.9 ± 0.3	0.49 ± 0.03

Values are mean ± s.d. using n = 5 independent experiments. The other experimental conditions were the same as those stated in **Experimental Section**.

<u>Supplementary Table S21.</u> The association and dissociation rate constants from ND-BLI for all tested constructs. ND-BLI data were derived from fits of individual sensorgrams (**Experimental Section**).

Nanopore Sensor	k_{on} ($M^{-1}s^{-1}$) $\times 10^{-4}$	k_{off} (s^{-1}) $\times 10^{2}$
**$(GGS)_2$**	1.9 ± 0.3	0.49 ± 0.03
**$O(GGS)_2$**	1.9 ± 0.2	0.46 ± 0.02
**$O(GGS)_4$**	2.0 ± 0.4	0.39 ± 0.04
**$O(GGS)_5$**	1.9 ± 0.2	0.37 ± 0.02
**$O(PA)_3$**	0.60 ± 0.08	1.8 ± 0.2
**$O\ (PA)_6$**	1.1 ± 0.2	1.2 ± 0.1
**$O\ (PA)_8$**	1.7 ± 0.1	1.1 ± 0.2

Values provide mean ± s.d. from independent experiments listed in above tables. The other experimental conditions were the same as those stated in **Experimental Section**.

<u>Supplementary Table S22.</u> The probability distribution of the three binding events of WDR5-$MLL4_{Win}$ interactions recorded with all nanopore sensors examined in this work. These events were differentiated by their WDR5-captured durations. Individual experimental values were derived using event-list histograms in ClampFit (Axon). Fits of event histograms were conducted in a semilogarithmic representation.[15] The maximum likelihood method[16, 17] and logarithm likelihood ratio (LLR) tests[18-20] were utilized to determine the best multi-exponential probability distribution model (**Experimental Section**).

Nanopore Sensor	P_1	P_2	P_3
**$O(GGS)_2$**	0.57 ± 0.05	0.29 ± 0.09	0.14 ± 0.03
**$O(GGS)_4$**	0.58 ± 0.06	0.28 ± 0.10	0.14 ± 0.02
**$O(PA)_3$**	0.73 ± 0.10	0.25 ± 0.03	0.02 ± 0.01
**$O(PA)_6$**	0.72 ± 0.08	0.26 ± 0.05	0.02 ± 0.01

Values represent mean ± s.d. obtained from independent experiments listed in above tables. The other experimental conditions were the same as those stated in **Experimental Section**.

Supplementary Table S23. The association and dissociation rate constants of the three binding events noted from dose response graphs. k_{on} values are the slopes of the linear fits in **Figure 3c** and **Figure 4c**. k_{off} values are the axis intercepts of the horizontal line fits in **Figure 3d** and **Figure 4d**.

Nanopore Sensor	$k_{on-1} \times 10^{-5}$ ($M^{-1}s^{-1}$)	$k_{on-2} \times 10^{-4}$ ($M^{-1}s^{-1}$)	$k_{on-3} \times 10^{-4}$ ($M^{-1}s^{-1}$)	k_{off-1} (s^{-1})	k_{off-2} (s^{-1})	k_{off-3} (s^{-1})
**O(GGS)$_2$**	1.1 ± 0.2	6.9 ± 0.4	3.5 ± 0.7	88 ± 4	9.4 ± 0.4	0.77 ± 0.03
**O(GGS)$_4$**	1.4 ± 0.2	7.9 ± 0.4	4.0 ± 0.5	86 ± 3	9.2 ± 0.5	0.78 ± 0.02
**O(PA)$_3$**	0.29 ± 0.09	1.0 ± 0.2	0.20 ± 0.04	220 ± 4	35 ± 1	0.79 ± 0.01
**O(PA)$_6$**	1.6 ± 0.2	5.1 ± 0.2	0.80 ± 0.04	170 ± 8	18 ± 1	0.80 ± 0.02

Values are mean ± s.e.m. from independent experiments listed in above tables. The other experimental conditions were the same as those stated in **Experimental Section**.

Supplementary Table S24. The association and dissociation rate constants obtained from WDR5-released and WDR5-captured event durations obtained with O(GGS)$_2$, O(GGS)$_4$, O(PA)$_3$, and O(PA)$_6$. The association rate constants of the short-lived events, k_{on-1}, were determined using the equation: $k_{on-1} = 1/(\tau_{on-1}[WDR5])$. The association rate constants of the medium-lived events, k_{on-2}, and long-lived events, k_{on-3}, were determined in the same way. The dissociation rate constants of the short-lived events were determined using the equation: $k_{off-1} = 1/\tau_{off-1}$. We used the same approach for determining the dissociation rate constants of the medium-lived events, k_{off-2}, and long-lived events, k_{off-3}.

Nanopore Sensor	$k_{on-1} \times 10^{-5}$ ($M^{-1}s^{-1}$)	$k_{on-2} \times 10^{-4}$ ($M^{-1}s^{-1}$)	$k_{on-3} \times 10^{-4}$ ($M^{-1}s^{-1}$)	k_{off-1} (s^{-1})	k_{off-2} (s^{-1})	k_{off-3} (s^{-1})
**O(GGS)$_2$**	1.4 ± 0.10	7.1 ± 1.8	3.6 ± 0.80	86 ± 6.1	9.2 ± 0.50	0.78 ± 0.06
**O(GGS)$_4$**	1.6 ± 0.20	7.8 ± 1.8	3.9 ± 1.0	87 ± 8.2	9.9 ± 0.30	0.77 ± 0.03
**O(PA)$_3$**	0.36 ± 0.10	1.3 ± 0.30	0.11 ± 0.02	210 ± 10	34 ± 1.1	0.79 ± 0.04
**O(PA)$_6$**	1.3 ± 0.20	4.8 ± 1.2	0.75 ± 0.05	170 ± 9.3	20 ± 2.1	0.77 ± 0.05

Values are mean ± s.d. obtained from independent experiments listed in above tables. The other experimental conditions were the same as those stated in **Experimental Section**.

Supplementary Table S25. Equilibrium dissociation constants, K_D, obtained from single-channel electrical recordings and ND-BLI measurements. ND-BLI data were derived from fits of individual sensorgrams (**Experimental Section**).

Nanopore Sensor	Single-molecule Electrical K_{D-1}(μM)	Single-molecule Electrical K_{D-2} (μM)	Single-molecule Electrical K_{D-3} (μM)	ND-BLI Optical K_D (μM)
**$(GGS)_2$**	NA	NA	NA	0.26 ± 0.03
**$O(GGS)_2$**	614 ± 20	130 ± 10	21 ± 5	0.25 ± 0.05
**$O(GGS)_4$**	544 ± 32	127 ± 13	20 ± 3	0.20 ± 0.05
**$O(GGS)_5$**	NA	NA	NA	0.20 ± 0.03
**$O(PA)_3$**	5833 ± 89	2615 ± 77	718 ± 21	3.01 ± 0.94
**$O\ (PA)_6$**	1308 ± 94	417 ±18	103 ± 11	1.10 ± 0.42
**$O\ (PA)_8$**	NA	NA	NA	0.62 ± 0.12

Values provide mean ± s.d.from independent experiments listed in above tables. NA stands for data not available due to the sensor performance. The other experimental conditions were the same as those stated in **Experimental Section**.

Supplementary references.

1. Imran, A.; Moyer, B. S.; Canning, A. J.; Kalina, D.; Duncan, T. M.; Moody, K. J.; Wolfe, A. J.; Cosgrove, M. S.; Movileanu, L., Kinetics of the multitasking high-affinity Win binding site of WDR5 in restricted and unrestricted conditions. *Biochem. J.* **2021,** *478* (11), 2145-2161.

2. Mayse, L. A.; Imran, A.; Larimi, M. G.; Cosgrove, M. S.; Wolfe, A. J.; Movileanu, L., Disentangling the recognition complexity of a protein hub using a nanopore. *Nature Commun.* **2022,** *13* (1), 978.

3. Imran, A.; Moyer, B. S.; Wolfe, A. J.; Cosgrove, M. S.; Makarov, A. A.; Movileanu, L., Interplay of Affinity and Surface Tethering in Protein Recognition *J. Phys. Chem. Lett.* **2022,** *13* (18), 4021-4028.

4. Imran, A.; Moyer, B. S.; Kalina, D.; Duncan, T. M.; Moody, K. J.; Wolfe, A. J.; Cosgrove, M. S.; Movileanu, L., Convergent Alterations of a Protein Hub Produce Divergent Effects Within a Binding Site. *ACS Chem. Biol.* **2022,** *17* (6), 1586-1597.

5. Mohammad, M. M.; Howard, K. R.; Movileanu, L., Redesign of a plugged beta-barrel membrane protein. *J. Biol. Chem.* **2011,** *286* (10), 8000-8013.

6. Thakur, A. K.; Movileanu, L., Real-Time Measurement of Protein-Protein Interactions at Single-Molecule Resolution using a Biological Nanopore. *Nature Biotechnol.* **2019,** *37* (1), 96-101.

7. Chen, X.; Zaro, J. L.; Shen, W. C., Fusion protein linkers: property, design and functionality. *Adv. Drug. Deliv. Rev.* **2013,** *65* (10), 1357-69.

8. Reddy Chichili, V. P.; Kumar, V.; Sivaraman, J., Linkers in the structural biology of protein-protein interactions. *Protein Sci.* **2013,** *22* (2), 153-67.

9. Sørensen, C. S.; Kjaergaard, M., Effective concentrations enforced by intrinsically disordered linkers are governed by polymer physics. *Proc. Natl. Acad. Sci. U S A* **2019,** *116* (46), 23124-23131.

10. Wriggers, W.; Chakravarty, S.; Jennings, P. A., Control of protein functional dynamics by peptide linkers. *Biopolymers* **2005,** *80* (6), 736-46.

11. Bryan, A. F.; Wang, J.; Howard, G. C.; Guarnaccia, A. D.; Woodley, C. M.; Aho, E. R.; Rellinger, E. J.; Matlock, B. K.; Flaherty, D. K.; Lorey, S. L.; Chung, D. H.; Fesik, S. W.; Liu, Q.; Weissmiller, A. M.; Tansey, W. P., WDR5 is a conserved regulator of protein synthesis gene expression. *Nucleic Acids Res.* **2020,** *48* (6), 2924-2941.

12. Guarnaccia, A. D.; Rose, K. L.; Wang, J.; Zhao, B.; Popay, T. M.; Wang, C. E.; Guerrazzi, K.; Hill, S.; Woodley, C. M.; Hansen, T. J.; Lorey, S. L.; Shaw, J. G.; Payne, W. G.; Weissmiller, A. M.; Olejniczak, E. T.; Fesik, S. W.; Liu, Q.; Tansey, W. P., Impact of WIN site inhibitor on the WDR5 interactome. *Cell Rep.* **2021,** *34* (3), 108636.

13. Dharmarajan, V.; Lee, J. H.; Patel, A.; Skalnik, D. G.; Cosgrove, M. S., Structural basis for WDR5 interaction (Win) motif recognition in human SET1 family histone methyltransferases. *J. Biol. Chem.* **2012,** *287* (33), 27275-89.

14. Movileanu, L.; Cheley, S.; Howorka, S.; Braha, O.; Bayley, H., Location of a Constriction in the Lumen of a Transmembrane Pore by Targeted Covalent Attachment of Polymer Molecules. *J. Gen. Physiol.* **2001,** *117* (3), 239-251.

15. Sigworth, F. J.; Sine, S. M., Data transformations for improved display and fitting of single-channel dwell time histograms. *Biophys. J.* **1987,** *52* (6), 1047-54.

16. Colquhoun, D.; Sigworth, F. J., Fitting and statistical analysis of single-channel records. In *Single-channel recording*, 2nd ed.; Sackmann, B., Neher,E., Ed. Plenum Press: New York, 1995; pp 483-587.

17. Colquhoun, D.; Hatton, C. J.; Hawkes, A. G., The quality of maximum likelihood estimates of ion channel rate constants. *J. Physiol.* **2003,** *547* (Pt 3), 699-728.

18. McManus, O. B.; Blatz, A. L.; Magleby, K. L., Sampling, Log Binning, Fitting, and Plotting Durations of Open and Shut Intervals From Single Channels and the Effects of Noise. *Pflugers Arch.* **1987,** *410* (4-5), 530-553.

19. McManus, O. B.; Magleby, K. L., Kinetic States and Modes of Single Large-Conductance Calcium-Activated Potassium Channels in Cultured Rat Skeletal-Muscle. *J. Physiol. (Lond.)* **1988,** *402*, 79-120.

20. Couoh-Cardel, S.; Hsueh, Y. C.; Wilkens, S.; Movileanu, L., Yeast V-ATPase Proteolipid Ring Acts as a Large-conductance Transmembrane Protein Pore. *Sci. Rep.* **2016,** *6*, 24774.
